# Trends in HIV care cascade engagement among diagnosed people living with HIV in Ontario, Canada: A retrospective, population-based cohort study

**DOI:** 10.1371/journal.pone.0210096

**Published:** 2019-01-04

**Authors:** James Wilton, Juan Liu, Ashleigh Sullivan, Beth Rachlis, Alex Marchand-Austin, Madison Giles, Lucia Light, Claudia Rank, Ann N. Burchell, Sandra Gardner, Doug Sider, Mark Gilbert, Abigail E. Kroch, Ontario HIV Epidemiology and Surveillance Initiative

**Affiliations:** 1 Data and Applied Science Impact, Ontario HIV Treatment Network, Toronto, Canada; 2 Public Health Ontario, Toronto, Canada; 3 Public Health Agency of Canada, Ottawa, Canada; 4 Division of Clinical Public Health, Dalla Lana School of Public Health, University of Toronto, Toronto, Canada; 5 Dignitas International, Toronto, Ontario, Canada; 6 Centre for Urban Health Solutions, Li Ka Shing Knowledge Institute, St. Michael’s Hospital, Toronto, Canada; 7 Department of Family and Community Medicine, St. Michael’s Hospital, Toronto, Canada; 8 Department of Family and Community Medicine and Dalla Lana School of Public Health, University of Toronto, Toronto, Canada; 9 Baycrest Health Sciences, Toronto, Canada; 10 Division of Biostatistics, Dalla Lana School of Public Health, University of Toronto, Toronto, Canada; 11 Clinical Prevention Services, British Columbia Centre for Disease Control, Vancouver, Canada; 12 School of Population and Public Health, University of British Columbia, Vancouver, Canada; The Ohio State University, UNITED STATES

## Abstract

**Background:**

The HIV cascade is an important framework for assessing systems of care, but population-based assessment is lacking for most jurisdictions worldwide. We measured cascade indicators over time in a population-based cohort of diagnosed people living with HIV (PLWH) in Ontario, Canada.

**Methods:**

We created a retrospective cohort of diagnosed PLWH using a centralized laboratory database with HIV diagnostic and viral load (VL) test records linked at the individual-level. Individuals enter the cohort with record of a nominal HIV-positive diagnostic test or VL test, and remain unless administratively lost to follow-up (LTFU, >2 consecutive years with no VL test and no VL test in later years). We calculated the annual percent of diagnosed PLWH (cohort individuals not LTFU) between 2000 and 2015 who were in care (≥1 VL test), on ART (as documented on VL test requisition) or virally suppressed (<200 copies/ml). We also calculated time from diagnosis to linkage to care and viral suppression among individuals newly diagnosed with HIV. Analyses were stratified by sex and age. Upper/lower bounds were calculated using alternative indicator definitions.

**Results:**

The number of diagnosed PLWH increased from 8,859 (8,859–11,389) in 2000 to 16,110 (16,110–17,423) in 2015. Over this 16-year period, the percent of diagnosed PLWH who were: in care increased from 81% (63–81%) to 87% (81–87%), on ART increased from 55% (34–60%) to 81% (70–82%) and virally suppressed increased from 41% (23–46%) to 80% (67–81%). Between 2000 and 2014, the percent of newly diagnosed individuals who linked to care within three months of diagnosis or achieved viral suppression within six months of diagnosis increased from 67% to 82% and from 22% to 42%, respectively. Estimates were generally lower for females and younger individuals.

**Discussion:**

HIV cascade indicators among diagnosed PLWH in Ontario improved between 2000 and 2015, but gaps still remain—particularly for younger individuals.

## Introduction

In recent years, the HIV cascade has become an important framework for monitoring HIV care, identifying gaps and informing/evaluating appropriate interventions [1]. The HIV cascade refers to the components of HIV diagnosis and care that people living with HIV (PLWH) progress through to achieve and maintain a suppressed viral load (VL). These components include testing and diagnosis, linkage to and retention in medical care, and initiation of and adherence to antiretroviral treatment (ART) [2]. With recent evidence supporting earlier initiation of ART to improve individual health and prevent HIV transmission to a partner [3,4], as well as a growing interest in the use of ART to reduce HIV incidence at a population-level [5–7], HIV policy and programming has increasingly focused on measuring and improving engagement in the HIV cascade. Indeed, the cascade framework has become the basis of several regional, national and international HIV policies, including the recent UNAIDS 90-90-90 strategy which calls for 90% of people living with HIV (PLWH) to be diagnosed, 90% of diagnosed PLWH to be on ART, and 90% of PLWH on ART to be virally suppressed by 2020 [8].

Although relatively simple in concept, there are several methodological challenges to measuring the cascade in ways that are representative of and comparable across jurisdictions [9–14]. These challenges include the lack of standardized cascade metrics, as well as limitations inherent to the varied data sources available. To understand and overcome these challenges, Medland and co-authors published a systematic review of cascade studies in 2015 [15]. In addition to proposing standardized metrics, the authors identified only six jurisdictions worldwide with optimal cascades; that is, “broad” cascades (i.e. include data on the number of infected/diagnosed PLWH through to the number suppressed) [10] constructed from population-based data sources (i.e. individual-level data collected across an entire population). These jurisdictions included New York City [16], King County in Washington State [17], British Columbia (BC) [18], Denmark [19], Georgia [20] and a study of 18 states and the District of Columbia representing approximately 40% of diagnosed PLWH in the US [21]. Further, only one of these studies used a cohort-based approach, which is optimal for longitudinal cascade measurement [10]. Instead, most published cascade analyses use clinical cohorts of PLWH who have already entered care and thus cannot be used to construct “broad” cascades [22–25], or extrapolate from non-population-based data sources and thus lack individual-level linkage from diagnosis to suppression (i.e. conduct analyses at the aggregate level) [26–30]. The lack of optimal data sources/approaches [10,15] means that accurate and comprehensive knowledge across the full continuum of care may be lacking for most jurisdictions worldwide.

Analysis of the HIV cascade in Ontario—Canada’s largest province (population 14.0 million) [31]—has been limited. Ontario has experienced the greatest burden of HIV in the country and about 40% of the ~80,000 HIV cases between 1985 and 2016 in Canada were diagnosed in Ontario [32,33]. In 2016, the HIV diagnosis rate per 100,000 people in Ontario (6.3) was comparable to the national average (6.4) [33]. Despite an overall decrease in the rate of HIV diagnoses and mortality among PLWH over time [34,35], approximately 800 to 900 HIV infections continue to be diagnosed in Ontario each year [34] and mortality remains higher for PLWH than the general population [35]. These data suggest there is room for improvement in HIV prevention and treatment in Ontario, and the provincial HIV/AIDS strategy launched in 2018 prioritizes cascade measurement to guide and evaluate HIV policy and programming [36]. However, analyses of cascade components to date have mostly been limited to a clinical cohort following approximately 25–30% of PLWH actively receiving medical care in the province [37], as well as a population-based administrative cohort of PLWH who have entered care that lacks information on viral suppression—a key component of the cascade [35,38,39]. While both of these data sources have important strengths, they are not optimal for assessing the full continuum of care at the level of Ontario’s population.

To improve our understanding of Ontario’s cascade, we created a population-based cohort of diagnosed PLWH (referred to as the Ontario HIV Laboratory Cohort) using a centralized laboratory database with HIV diagnostic and viral load (VL) test records linked at the individual-level. In this paper, we assess cascade indicators over time and by sex and age among diagnosed PLWH in the Ontario HIV Laboratory Cohort. Our primary objectives were to measure trends in the annual proportion of diagnosed PLWH who were in care, on ART and virally suppressed. We also aimed to measure time from diagnosis to linkage to care and viral suppression among individuals who were newly diagnosed with HIV in Ontario.

## Methods

### Setting

Ontario is Canada’s most populous province [31]. Access to medically necessary services (including HIV medical care visits and laboratory testing) are free for those living in Ontario who are eligible (e.g. Canadian citizens, permanent residents) and registered with the province’s health care plan. However, in Ontario, there is no universal coverage of ART medications. Individuals can obtain coverage through private health insurance or may be eligible for government-sponsored programs [40]. However, private and public coverage is not always full and often requires some form of payment to cover deductibles/co-payments [41].

### Data sources

#### Public Health Ontario Laboratory HIV datamart and Ontario HIV Laboratory Cohort

The Ontario HIV Laboratory Cohort is a retrospective, population-based cohort of diagnosed PLWH in Ontario, Canada. This cohort was created using the HIV datamart at Public Health Ontario Laboratory (PHOL). The HIV datamart and creation of the Ontario HIV Laboratory Cohort are described below.

The HIV datamart was developed using HIV clinical laboratory databases housed at PHOL. All HIV diagnostic and VL testing conducted by health care providers in Ontario is done by PHOL. Centralized databases contain information on test results and information documented by the ordering provider on test requisition/surveillance forms. The diagnostic test requisition collects information on sex, age and HIV exposure category while the VL test requisition collects information on sex, age, ART medications and most recent CD4 count. A follow-up surveillance form (the Laboratory Enhancement Program, LEP, questionnaire) is sent to an ordering provider if a diagnostic test result is HIV-positive in order to collect further information on the diagnosed individual. This includes information collected on the diagnostic test requisition (e.g. exposure category), as well as information not collected on the requisition (e.g. race/ethnicity and country of birth). The LEP form was introduced in 1999 and race/ethnicity and country of birth were added to the questionnaire in 2009. The previously separate diagnostic and VL databases were recently integrated by Public Health Ontario and records linked at the individual-level to create a single population-based data source (hereafter referred to as the “HIV datamart”). Of note, the majority of HIV diagnostic testing in Ontario is conducted nominally (i.e. using the name of the person tested), although non-nominal forms of testing (i.e. using anonymous or coded identifiers) are permitted. VL testing in Ontario is conducted as part of routine care for people already diagnosed with HIV. While anonymous VL testing has been permitted as of July 1^st^ 2015, all VL tests in the database were nominal as of the end of 2015.

We used the HIV datamart to create a cohort of diagnosed PLWH in Ontario for retrospective measurement of cascade indicators. Individuals in the datamart enter the cohort with first record of a nominal HIV-positive diagnostic test or VL test. Non-nominal HIV-positive diagnoses are excluded due to insufficient identifying information to allow linkage to other diagnostic/VL tests (thus precluding the ability to identify possible duplication in the nominal data, as well as measure subsequent engagement in care). However, while non-nominal tests are excluded, individuals diagnosed non-nominally enter the cohort when they link to care and receive a nominal VL test. As such, individuals with a VL test only (no linked HIV-positive diagnostic test) are included in the cohort to capture individuals diagnosed non-nominally, as well as previously diagnosed individuals migrating into the province. All individuals who enter the cohort are defined as a person with diagnosed HIV.

Individuals in the cohort remain unless administratively lost-to-follow up (LTFU), defined as having had no VL test for >2 consecutive years and no VL test in later years. The LTFU rule was applied to indirectly censor for death and migration out of the province (out-migration), as this information was lacking from our laboratory databases. Importantly, participants assessed as LTFU are not permanently censored and, as time progresses, these individuals can re-enter the cohort with subsequent record of a VL test. We selected 2 years for the LTFU criteria given that this would capture most individuals experiencing a known gap in care in our cohort (i.e. no VL test in ≥1 year but record of a VL test in a later year). The duration of this LTFU criteria is similar to what has been used elsewhere [18,37]. We define cohort individuals who are not LTFU as diagnosed people *living* with HIV (PLWH) in Ontario.

#### Newly diagnosed sample

We also used the datamart to create a subset of individuals who were newly diagnosed with HIV in Ontario (i.e. were not initially diagnosed elsewhere and then moved to Ontario) in order to measure longitudinal cascade indicators (i.e. time from diagnosis to linkage to care and viral suppression). This newly diagnosed subset includes individuals with record of a nominal HIV-positive diagnostic test and excludes individuals with 1) a VL test only (i.e. no linked HIV-positive diagnostic test—as this precludes measurement of time from HIV diagnosis) and 2) evidence of having received an HIV-positive diagnosis prior to their first nominal HIV-positive diagnostic test record in Ontario (i.e. record of a CD4 or detectable VL test before diagnosis, or a first VL test after diagnosis that was suppressed).

### Indicators and definitions

Our cascade indicators are summarized in Table 1. We selected and defined indicators based on available data, a review of the literature, and expert opinion—with most being similar to those recommended by Medland et al in a recent systematic review [15]. In contrast to Medland et al [15], we used the term “in care” instead of “retention in care”, as we felt this is more reflective of what the indicator is measuring (i.e. while our dataset is longitudinal, the measure of ≥1 VL test in a given year is cross-sectional in nature and doesn’t track an individual’s retention longitudinally). In addition to the elements recommended by Medland et al, we also measured time from diagnosis to viral suppression, as done by others [42,43]. While linkage to care is defined as the percent of individuals who linked to care within 3 months of diagnosis [15], there is no recommended threshold for time from diagnosis to suppression. In the presentation of our results, we focus on the percent suppressed within 6 months. Of note, the national HIV/AIDS strategy in the United States shortened the threshold for their linkage to care indicator from 3 months to 1 month in 2015 [44]. Therefore, we also include the proportion linked to care within 1 month of diagnosis in respective figures.

**Table 1:** Indicators used to monitor cascade engagement in the Ontario HIV Laboratory Cohort and most recent estimates for each indicator.

Indicator	Definition (numerator)	Denominator used for calculating percent of individuals per calendar year	2015 cohort estimates
Diagnosed PLWH			
Main	Confirmed nominal HIV-positive diagnostic test and/or ≥1 VL test, and not administratively lost to follow-up after 2 years	—	16,110
Upper	Confirmed HIV-positive diagnostic test (nominal or **non-nominal**) and/or ≥1 VL test, and not administratively lost to follow-up after **3 years**	—	17,423
In care			
Main	≥ 1 VL test in given year	Diagnosed PLWH (Main)	87.3%
Lower	≥ 1 VL test in given year	Diagnosed PLWH (Upper)	80.7%
On ART ^a^			
Main	Documented on ART, or ART status not documented and suppressed, on **last** VL test in a given year	Diagnosed PLWH (Main)	81.1%
Upper	Documented on ART, or ART status not documented and suppressed, on **any** VL test in a given year	Diagnosed PLWH (Main)	82.0%
Lower	Documented on ART, or ART status not documented and suppressed, on **all** VL tests in a given year	Diagnosed PLWH (Upper)	69.9%
Virally suppressed ^a^			
Main	VL <200 copies/mL on **last** VL test in a given year	Diagnosed PLWH (Main)	79.5%
Upper	VL <200 copies/mL on **any** VL test in a given year	Diagnosed PLWH (Main)	80.8%
Lower	VL <200 copies/mL on **all** VL tests in a given year	Diagnosed PLWH (Upper)	67.4%
Virally suppressed (among those on ART)			
Main	VL <200 copies/mL, and known on ART or ART status not documented, on **last** VL test in a given year	Documented on ART on last VL, or ART status not documented, on **last** VL test in a given year	94.4%
Upper	VL <200 copies/mL, and known on ART or ART status not documented, on **any** VL test in a given year	Documented on ART, or ART status not documented, on **any** VL test in a given year	95.1%
Lower	VL <200 copies/mL, and known on ART or ART status not documented, on **all** VL tests in a given year	Documented on ART, or ART status not documented, on **all** VL tests in a given year	90.9%
Newly diagnosed			
Main	Confirmed nominal HIV-positive diagnostic test and no evidence of diagnosis prior to nominal HIV-positive diagnostic test	—	473 (2014 estimate)
Time to linkage to care			
Main	First VL test ≤ 3 months after diagnosis	Newly diagnosed (Main)	81.8% (2014 estimate)
Time to suppression			
Main	First suppressed VL (<200 copies/ml) ≤ 6 months after diagnosis	Newly diagnosed (Main)	41.6% (2014 estimate)

ART status documented by providers on VL test requisitions and missing from 17–20% of requisitions each year. Assumptions for requisitions with missing ART data differ by indicator. Bold text highlights differences between main and upper/lower definitions. 2015 estimates not shown for newly diagnosed indicators to avoid truncation bias.

^a^ Conditional estimates were also calculated for these indicators using the number ‘in care’ in the denominator. VL = viral load. ART = antiretroviral treatment. PLWH = people living with HIV.

Medland et al recommend that cascade indicators be presented as both the percent of all PLWH in a jurisdiction (diagnosed and undiagnosed) as well as all diagnosed PLWH [15]. In our analysis, we only present cascade indicators as the percent of diagnosed PLWH, as the total HIV-infected population in the province was not measurable in our cohort and modeling estimates over this time period are currently under development in Ontario. To quantify attrition at each cascade step and facilitate comparison to the third UNAIDS 90-90-90 estimate, we also calculated conditional estimates of those “on ART” and “virally suppressed” by limiting the denominator to individuals who had already achieved the previous indicator(s) (i.e. were already “in care” or “on ART”).

For each cascade indicator, we created a “main” definition based on the most commonly used and recommended approach. In keeping with recommendations to present a range of plausible estimates (as opposed to what may be artificially precise single estimates) [14], we also calculated “upper” and “lower” bounds. These upper/lower bounds were calculated using alternative assumptions/definitions for each indicator, where possible. Alternative assumptions/definitions were employed to improve our ability to compare across studies (as there is no consistent standard for cascade metrics across the literature) and to accommodate possible biases due to inherent aspects unique to our data source. Of note, for our alternative (“upper”) definition of the number of diagnosed PLWH (cohort individuals not LTFU), we altered our eligibility criteria to include non-nominal HIV-positive diagnoses and extended the LTFU criteria from two to three years. We used this “upper” diagnosed PLWH definition in the denominator to calculate “lower” estimates of the percent “in care”, “on ART” and “virally suppressed”. For our main definition of “virally suppressed”, we used the most recent VL test in a given year, which is the approach most commonly used [16,17,21] and recommended [45] by others, but is not specified by Medland et al [15].

Our “on ART” indicator was based on documentation of specific antiretroviral medications by the ordering provider on VL test requisition forms. This information was missing for approximately 17–20% of requisitions each year. To address these missing data in our analysis, we made conservative assumptions regarding ART status (i.e. on or off ART) for requisitions with missing information. In order to calculate conservative estimates, our assumptions differed depending on whether “on ART” was used in the numerator or denominator of the indicator calculated. When “on ART” was in the numerator (to calculate the percent of diagnosed PLWH who were on ART), requisitions missing information were only assumed to be on ART if the VL was suppressed. When “on ART” was in the denominator (to calculate the percent of PLWH on ART who were suppressed), all requisitions with missing ART information were assumed to be on ART.

### Analysis

We restricted our analyses to the period from January 1, 2000 to December 31, 2015. Although VL testing was implemented in 1996, it took several years to become a routine part of HIV care and thus serve as an accurate proxy for linkage to and engagement in care [46].

We used data from the Ontario HIV Laboratory Cohort to measure trends in the annual percent of diagnosed PLWH who were in care, on ART and virally suppressed, as well as conditional estimates for the on ART and virally suppressed indicators. Cohort participants were included in the analyses of annual cascade measures until administratively LTFU. Individuals were assumed to be off ART and unsuppressed if they did not have a VL test in a given year. Individuals who re-entered the cohort after being LTFU were counted as being a diagnosed PLWH who did not meet any of the cascade indicator definitions for the years in which they were LTFU.

We used data from the newly diagnosed subset to measure trends in the annual percent of newly diagnosed individuals who linked to care within three months of diagnosis (i.e. linkage to care) and achieved viral suppression within six months of diagnosis. Among those with record of a VL test or suppressed VL, we also calculated the median number of days from diagnosis-to-care and diagnosis-to-suppression, respectively. To minimize truncation bias, we excluded individuals newly diagnosed in 2015 from the diagnosis-to-care analyses and those diagnosed in 2014 and 2015 from the diagnosis-to-suppression analyses, given that individuals diagnosed in these years may not have had sufficient time to reach these endpoints by end of 2015.

We assessed cascade indicators by sex and age, both of which are collected on the diagnostic and VL test requisition forms and missing for less than 1% of the cohort. We did not explore indicators by race/ethnicity or HIV exposure category as this information was missing for approximately half of participants or more. This missing information was due to a combination of factors, including providers not filling out diagnostic requisition forms and race/ethnicity not being collected on the LEP prior to 2009. In addition, race/ethnicity and HIV exposure category are not collected on VL test requisition forms and are therefore missing for participants with no linked HIV diagnostic test (approximately 25% of participants).

All analyses were descriptive and no formal statistical testing across time or populations was conducted. We considered this reasonable as our data is population-based (not a random sample) [47] and the large sample size would mean even small differences are statistically significant [48].

### Ethical approval

This applied research study was approved by the Ethics Review Board at Public Health Ontario.

## Results

### Cohort creation and follow-up

**Fig 1:**
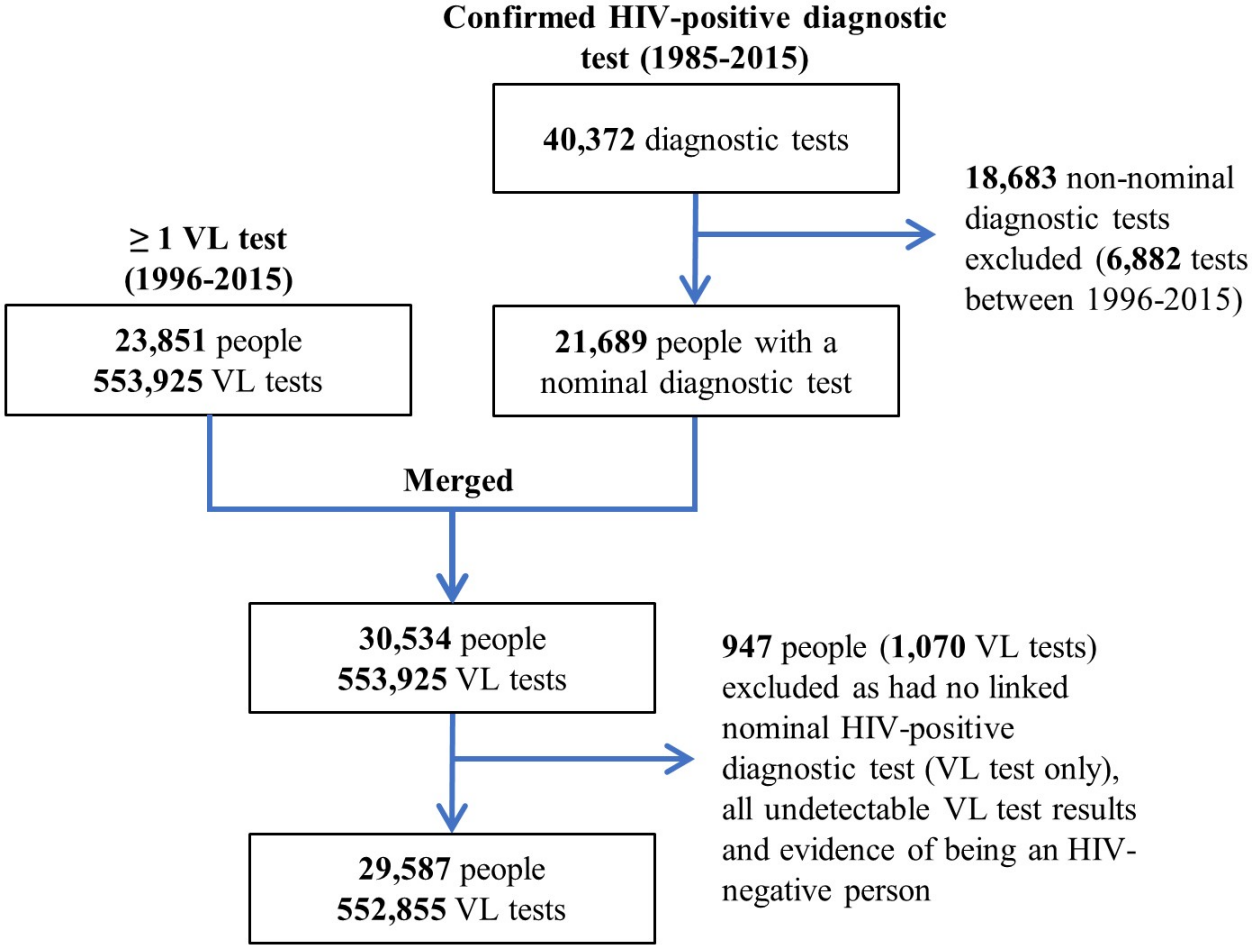
Flow diagram for the creation of the Ontario HIV Laboratory Cohort from the Public Health Ontario Laboratory HIV datamart. Non-nominal forms of testing include the use of coded or completely anonymous identifiers. Evidence of being an HIV-negative person = record of a nominal HIV-negative diagnostic test after, on the same day as, or within 30 days before last undetectable viral load test. VL = viral load.

As of 2015, there were a cumulative total of 40,372 confirmed HIV-positive diagnostic test records (1985–2015) and 23,851 unique individuals with a record of ≥1 VL test (1996–2015) in the HIV datamart (Fig 1). Of the HIV-positive diagnostic tests, 18,683 (46.3%) were conducted non-nominally and excluded from the cohort. Of note, the percent of diagnostic tests that were non-nominal decreased from 48.9% in 2000 to 15.0% in 2015 (S1 Table).

**Fig 2:**
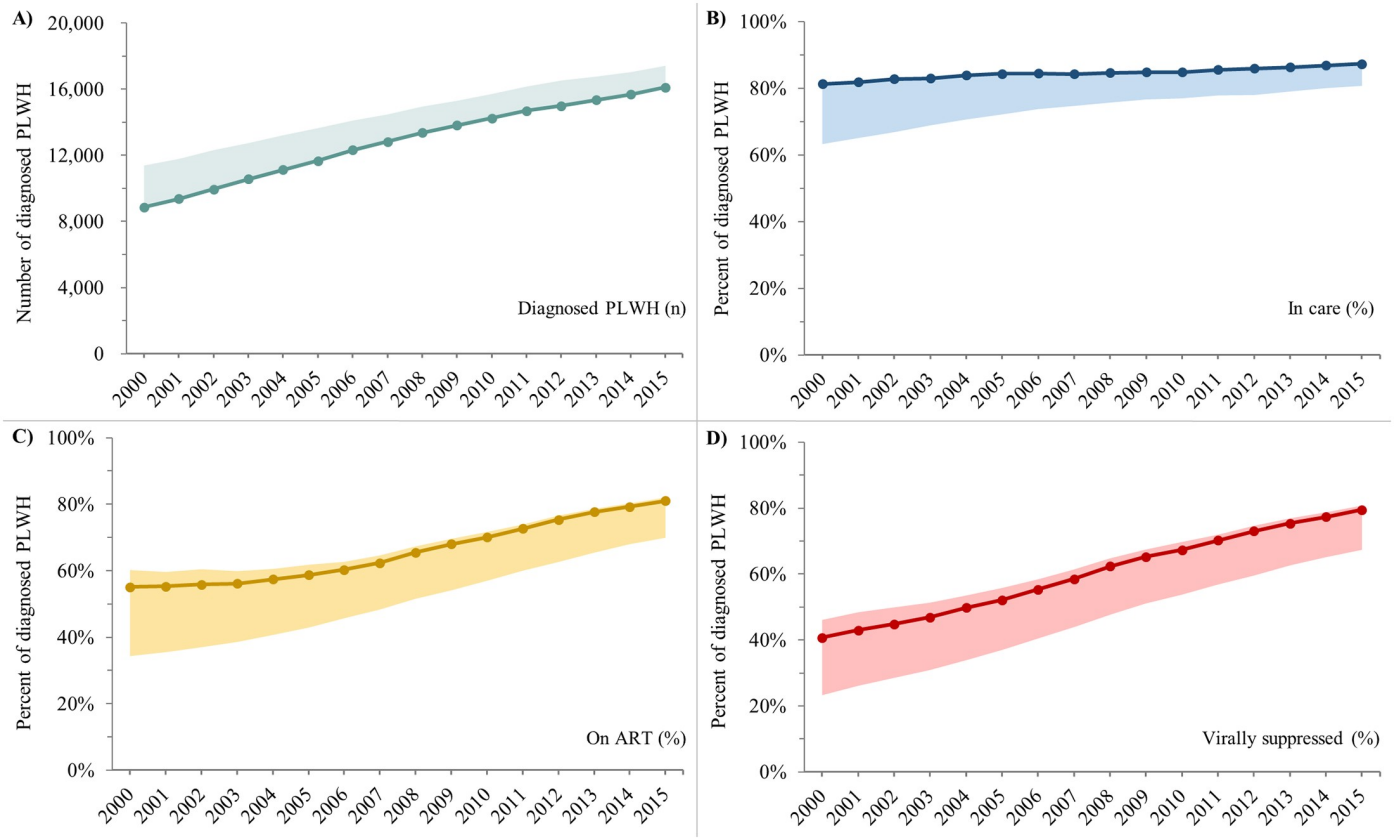
Trends in the number of diagnosed PLWH and the percent who were in care, on ART, and virally suppressed, Ontario HIV Laboratory Cohort, 2000–2015. A) Number of diagnosed PLWH. B) Percent of diagnosed PLWH who were in care. C) Percent of diagnosed PLWH who were on ART. D) Percent of diagnosed PLWH who were virally suppressed. Solid lines represent “main” estimates and shaded areas represent “upper” and/or “lower” bounds. See Table 1 for indicator definitions. PLWH = people living with HIV. ART = antiretroviral treatment.

Overall, 29,587 unique individuals with a nominal HIV-positive diagnostic test and/or ≥1 VL test were included in the cohort. As of the end of 2015, these individuals had been followed for a cumulative total of 229,302 person-years and 552,855 VL tests. Between 2000 and 2015, the number of diagnosed PLWH (cohort individuals not LTFU) increased from 8,859 (upper bound: 11,389) to 16,110 (upper bound: 17,423) (Fig 2A).

### Cohort sociodemographic profile

In 2015, the majority of the 16,110 diagnosed PLWH in the cohort were male (79.6%) or 45 years of age or older (62.6%) (Table 2). The percent of diagnosed PLWH who were 45 years of age or older doubled from 29.1% in 2000 to 62.6% in 2015, while the percent who were female increased from 15.0% in 2000 to 20.0% in 2008 and has since remained relatively stable (Tables A and B in S2 Table).

**Table 2:** Sociodemographic characteristics of diagnosed PLWH in the Ontario HIV Laboratory Cohort, 2015 (N = 16,110).

Characteristic	Diagnosed PLWH	
	n	%
**Sex (where known)**		
Female	3,257	20.4%
Male	12,724	79.6%
**Age (where known)**		
<25	469	2.9%
25–34	2,009	12.5%
35–44	3,529	22.0%
45–54	5,737	35.7%
55+	4,329	26.9%
**Period of HIV diagnosis (where known)**		
Prior to 1996	1,548	13.7%
1996–2000	1,325	11.7%
2001–2005	2,230	19.7%
2006–2010	2,916	25.7%
2011–2015	3,306	29.2%
**Race/ethnicity (where known)**		
White	1,367	51.4%
Black	714	26.9%
Latin American	163	6.1%
East/Southeast Asian	145	5.5%
South Asian	108	4.1%
Indigenous	75	2.8%
Arab/West Asian	46	1.7%
Other/mixed	40	1.5%
**HIV exposure category (where known)**		
MSM	3,787	46.4%
PWID	881	10.8%
Heterosexual	1,515	18.5%
HIV-endemic	1,334	16.3%
No identified risk factor(s)	737	4.6%
**Missing**^a^		
Sex	129	0.8%
Age	37	0.2%
Period of diagnosis	4,785	29.7%
Race/ethnicity	13,452	84.5%
Exposure category	7,941	49.3%

Diagnosed PLWH are cohort participants not lost to follow-up. All characteristics mutually exclusive, except for MSM and PWID exposure categories.

^a^ Reasons for missing data include 1) ordering providers not filling out requisition forms, 2) race/ethnicity only being collected from 2009 onwards, and 3) information on these characteristics only being collected on diagnostic forms and therefore missing for the 29.7% of participants in 2015 with a VL test only (no linked diagnostic test). MSM = men who have sex with men. PWID = people who use injection drugs. PLWH = people living with HIV.

Date of diagnosis, race/ethnicity and HIV exposure category were missing for a large proportion of participants (Table 2). Where this information was known, the majority of individuals in 2015 were diagnosed between 2006 and 2015 (54.9%), the most common race/ethnicity was White (51.4%) followed by Black (26.9%), and the most common HIV exposure category was men who have sex with men (46.4%) followed by heterosexual (18.5%).

### Cascade indicators

**Fig 3:**
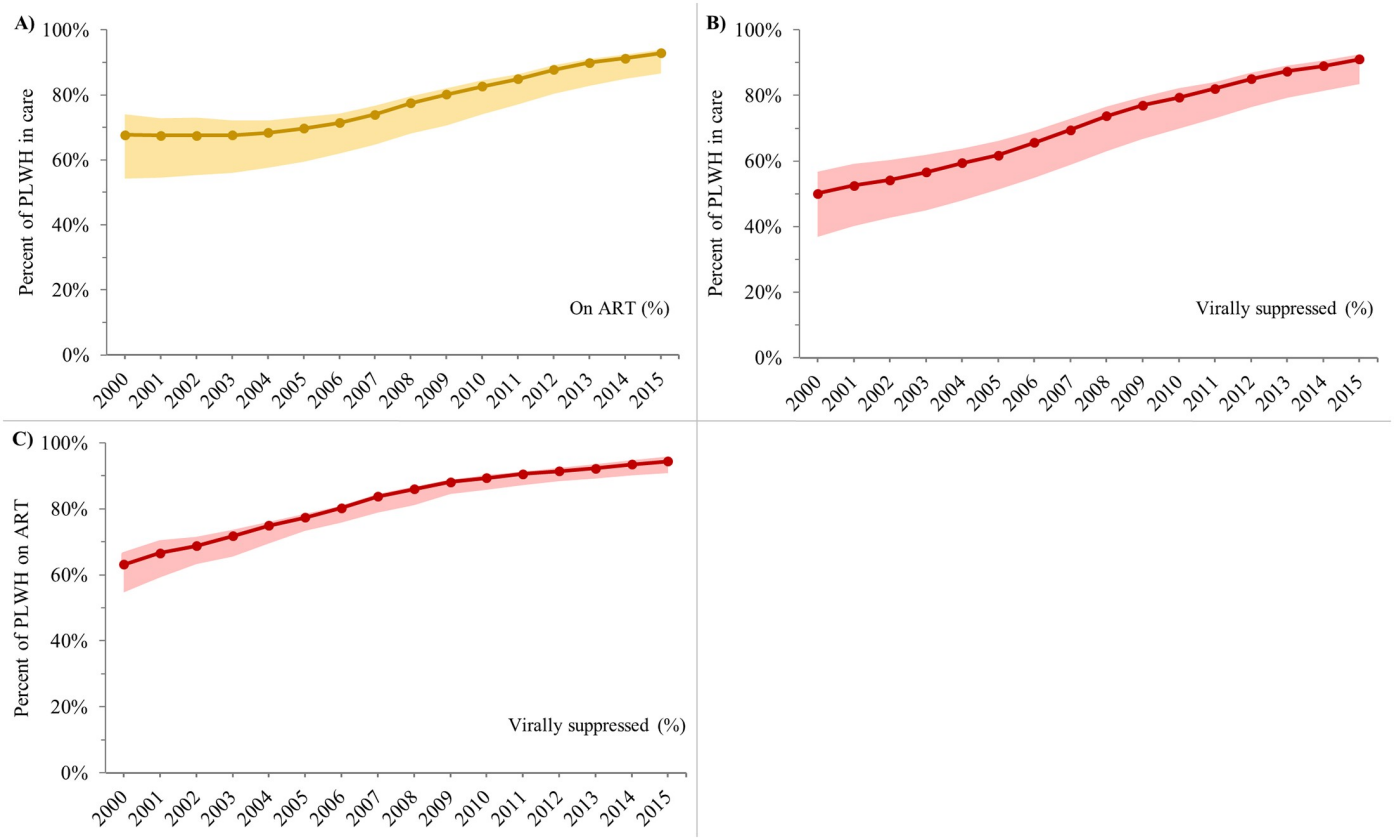
Trends in conditional cascade indicators, Ontario HIV Laboratory Cohort, 2000–2015. A) Percent of diagnosed PLWH in care who were on ART. B) Percent of diagnosed PLWH in care who were virally suppressed. C) Percent of diagnosed PLWH on ART who were virally suppressed. Solid lines represent “main” estimates and shaded areas represent “upper” and/or “lower” bounds. See Table 1 for indicator definitions. PLWH = people living with HIV. ART = antiretroviral treatment.

**Fig 4:**
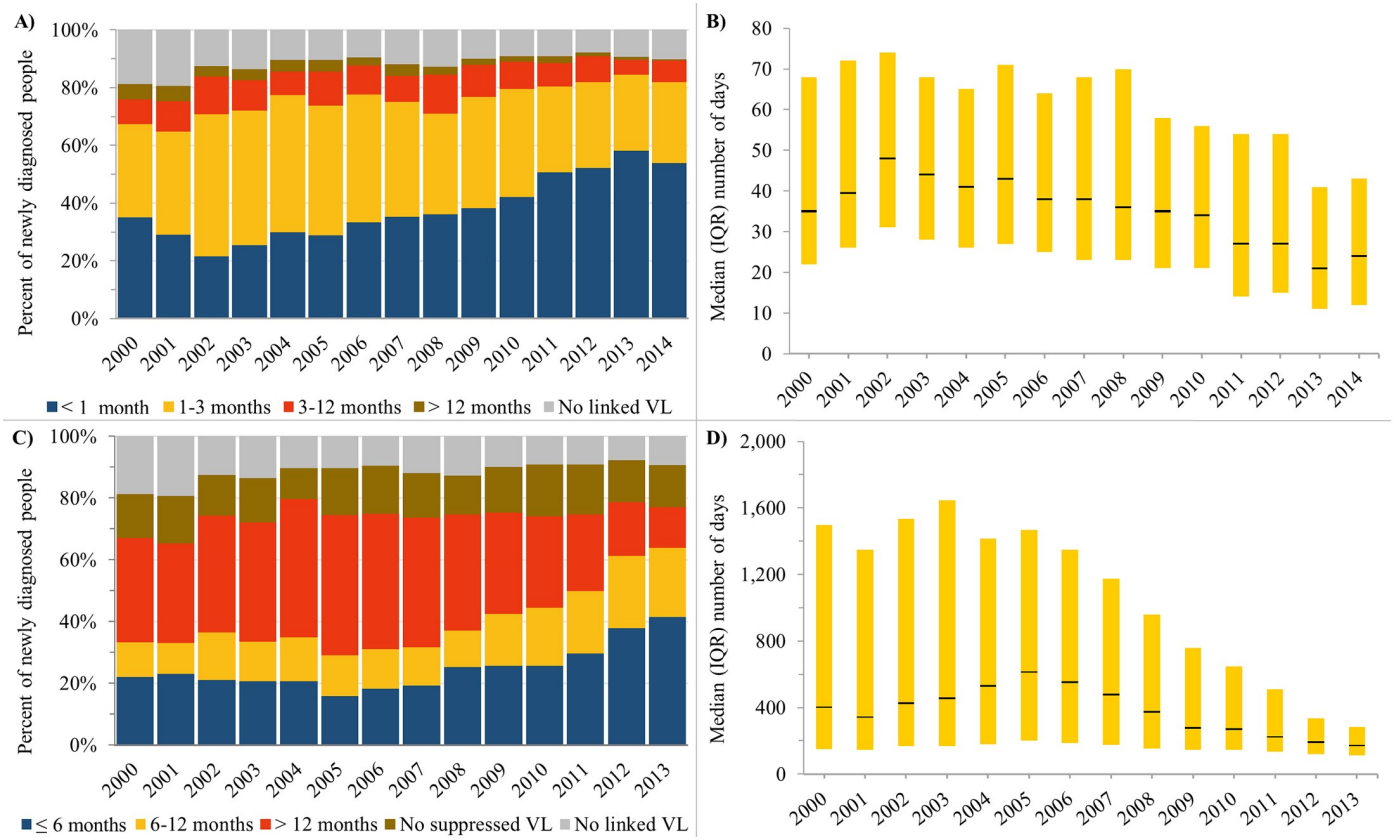
Trends in time from HIV diagnosis to linkage to care and viral suppression among individuals newly diagnosed with HIV in Ontario, 2000-2013/2014. A) Percent of newly diagnosed individuals who linked to care within a certain number of months after HIV diagnosis. B) Median (IQR) number of days from HIV diagnosis to linkage to care. C) Percent of newly diagnosed individuals who achieved viral suppression within a certain number of months after HIV diagnosis. D) Median (IQR) number of days for HIV diagnosis to viral suppression. In Fig B and D, yellow bar indicates interquartile range and black line indicates median.

**Fig 5:**
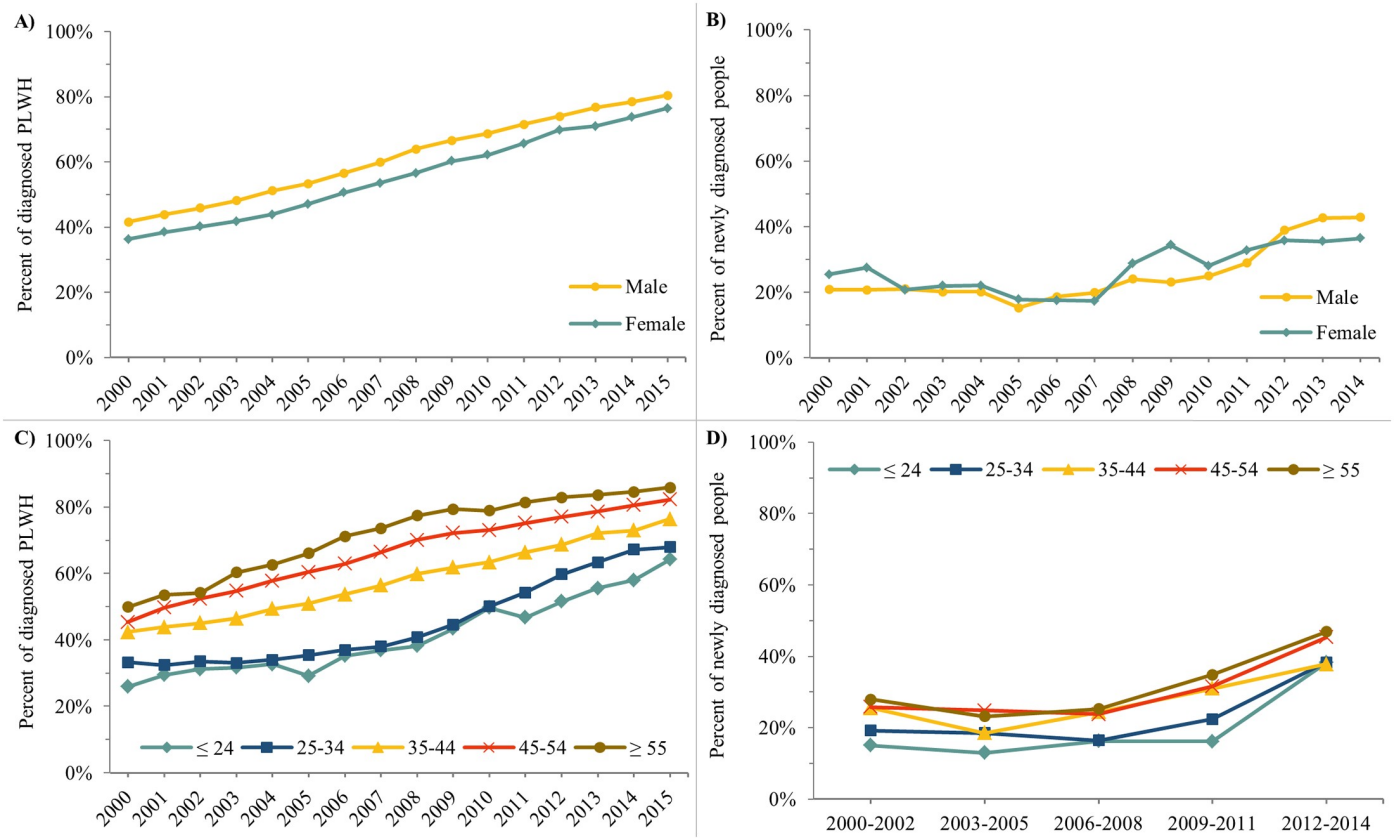
Trends in viral suppression indicators by sex and age category (main estimates only), Ontario HIV Laboratory Cohort, 2000-2014/15. A) Percent of diagnosed PLWH who were virally suppressed by sex. B) Percent of newly diagnosed individuals who achieved viral suppression within 6 months of HIV diagnosis by sex. C) Percent of diagnosed PLWH who were virally suppressed by age category. D) Percent of newly diagnosed individuals who achieved viral suppression within 6 months of HIV diagnosis by age category. In Fig D, percents averaged over three years to reduce year-to-year variation due to small counts. In Fig 5B and 5D, individuals with no VL are included in the denominator.

Indicator estimates for the most recent years of analysis (2014/2015) are summarized in Table 1. Trends over time and breakdowns by sex and age are summarized below and in Figs 2–5. Data underlying all figures can be found in the Tables in S1 Supporting Information.

#### Main indicators among diagnosed PLWH

The percent of diagnosed PLWH who were in care, on ART and virally suppressed all increased over time. Between 2000 and 2015, the percent who were in care increased from 81.3% (lower bound: 63.2%) to 87.3% (lower bound: 80.7%) (Fig 2B), the percent who were on ART increased from 55.1% (lower and upper bounds: 34.3%-60.2%) to 81.1% (69.9%-82.0%) (Fig 2C) and the percent who were virally suppressed increased from 40.7% (23.3%-46.1%) to 79.5% (67.4%-80.8%) (Fig 2D).

#### Conditional indicators among diagnosed PLWH

Between 2000 and 2015, the percent of PLWH in care who were on ART increased from 67.7% (54.3%-74.1%) to 92.8% (86.6%-93.9%) (Fig 3A) and who were suppressed increased from 50.1% (36.9%-56.7%) to 91.1% (83.5%-92.5%) (Fig 3B). Over the same time period, the percent of PLWH on ART who were suppressed increased from 63.2% (54.7%-66.2%) to 94.4% (90.9%-95.1%) (Fig 3C).

#### Longitudinal indicators among newly diagnosed individuals

Between 1996 and 2015, 19,386 individuals were diagnosed nominally and eligible for inclusion in the newly diagnosed subset (Figure A in S2 Supporting Information shows flow diagram). Of these, 5,976 (30.8%) had evidence of being previously diagnosed and were excluded. A total of 8,173 were newly diagnosed from 2000 onwards and included in our analyses of time from diagnosis to care and viral suppression. Between 2000 and 2015, the median annual number of newly diagnosed individuals was 529 (range: 368–599) (Table A in S2 Supporting Information).

Time from diagnosis to linkage to care and viral suppression both improved over time (Fig 4). The percent of newly diagnosed individuals who linked to care within 3 months of diagnosis increased from 67.4% in 2000 to 81.8% in 2014 (Fig 4A). The percent who achieved viral suppression within 6 months of diagnosis was relatively stable at approximately 20% between 2000 and 2007, and then increased to 41.4% by 2013 (Fig 4C).

The percent of newly diagnosed individuals who did not have record of a VL test or suppressed VL was relatively stable over time at approximately 10% and 15%, respectively. Among newly diagnosed individuals with record of these endpoints, time from diagnosis-to-care and diagnosis-to-suppression decreased over time. The median (interquartile range, IQR) number of days from diagnosis-to-care peaked at 48 (31–74) in 2002 and then decreased to 24 (12–43) days in 2014 (Fig 4B). The median (IQR) number of days from diagnosis-to-suppression peaked at 614 (202–1,469) in 2005 and then decreased to 172 (112–285) days in 2013 (Fig 4D).

#### Cascade indicators by sex and age

Indicators related to viral suppression are shown by sex and age in Fig 5 (main estimates only). All other indicators are shown by sex and age in the Tables in S3 Supporting Information.

Cascade estimates among diagnosed PLWH were generally consistently higher for males compared to females, but these differences were relatively minor and rarely exceeded seven absolute percentage points. In 2015, 80.4% of diagnosed male PLWH were virally suppressed compared to 76.5% of females (Fig 5A). In contrast, the percent of newly diagnosed individuals who linked to care within 3 months of diagnosis was relatively similar by sex and not consistently higher for males or females (Fig 5B).

Differences by age were more noticeable, with indicator estimates generally consistently higher for older age groups. In 2015, the percent of diagnosed PLWH who were virally suppressed was 64.2%, 67.9%, 76.4%, 82.2%, and 85.9% among those aged <25, 25–34, 35–44, 45–54, and 55+, respectively (Fig 5C). Across the same age gradient, the percent of newly diagnosed individuals in 2012–2014 who were virally suppressed within 6 months of diagnosis was 38.3%, 38.3%, 37.8%, 45.4%, and 46.9%, respectively (Fig 3D).

## Discussion

We observed improved engagement in the HIV cascade among diagnosed PLWH in Ontario, Canada between 2000 and 2015. Our study is one of few published efforts to have measured the cascade using a population-based data source with individual-level linkage from diagnosis to suppression [15], and to our knowledge only the second of these to use a cohort-based approach [15,18], despite such data sources/approaches being recommended as optimal for cascade measurement [10,15]. In our study, the “linked to care” and “in care” indicators were both relatively high across the 16-year study period and increased only slightly, while there were more dramatic increases in the “on ART”, “virally suppressed”, and time from diagnosis-to-suppression indicators. These trends are similar to those observed in other population-based HIV cascade studies [16,18]. Improvements in cascade indicators over time are likely due to a combination of factors, such as better access to care and ART, availability of ART regimens that are more effective and easier to take, changes to ART guidelines recommending earlier initiation of treatment and opposing treatment interruptions, the success of care and treatment initiatives, and/or changes in the diagnosed population over time.

Our results fill an important gap in HIV cascade knowledge in Ontario and complement other related studies. The Ontario HIV Treatment Network Cohort Study (OCS) follows over 4000 PLWH actively receiving medical care from specialty HIV clinics across the province and includes approximately a quarter of all PLWH receiving care in Ontario [49,50]. Our results are similar to those observed in the OCS: a relatively stable percent in care, increases in ART use beginning in the mid-2000s and a relatively constant increase in the percent who are virally suppressed [37]. Another study team in Ontario has developed an administrative cohort of PLWH using population-based data on physician billing claims housed at the Institute of Clinical Evaluative Sciences (ICES). Between 2009–2012, the majority of individuals in this cohort accessed HIV-related care through family physicians (55%) followed by HIV specialists (36%), while the remainder received little-to-no usual care (9%) [38]. Our estimate of about 15% of diagnosed PLWH who were not in care is higher than the 9% reported through the ICES cohort, which is expected as our cohort includes those who never enter care after diagnosis.

Despite high and improving cascade estimates, we still identified gaps where improvement is needed. In 2015, 13% of diagnosed PLWH were not in care, 7% were in care but not on ART and 6% were on ART but not virally suppressed. These gaps were larger if the lower bounds for these indicators were considered. In addition, 18% were not linked to care within three months of diagnosis and 59% did not achieve viral suppression within six months.

Cascade indicators were also generally lower for younger individuals, as observed in a clinical HIV cohort in Ontario (the OCS) [37,51] and other jurisdictions [16,30,52]. While our analyses did not adjust for time since diagnosis (many older individuals may have had more time to progress through the cascade stages), others in Ontario have reported that the association between older age and higher indicator estimates remains after adjustment for this potential confounder [37]. Further, we observed more timely viral suppression among individuals who were newly diagnosed with HIV at older ages, an analysis which inherently adjusts for time since diagnosis. Possible barriers to HIV care and treatment among youth include stigma, housing instability, transportation, mental health and substance use issues, difficulty accessing appropriate support services, and challenges in transitioning from pediatric to adult care [53–55].

In contrast to studies from the United States [16,30], but similar to an analysis from the Canadian province of BC [52], our indicator estimates among diagnosed PLWH were consistently slightly lower for females. Importantly, however, we did not explore cascade estimates by sub-populations of males and females, and other studies have demonstrated lower engagement in care among heterosexual males compared to men who have sex with men [16,37,51,52]. Interestingly, we did not observe a consistent difference by sex in our longitudinal indicators among newly diagnosed cases. This may be due to dissimilarities between prevalent and newly diagnosed cases, or due to bias introduced by excluding individuals with a VL test only from the newly diagnosed sample. The latter may be true as many of these excluded individuals were likely non-nominally diagnosed individuals, and non-nominal diagnoses in Ontario are more likely to be MSM (Table A in S3 Table) who, as already mentioned, are a population that tends to experience better engagement in the cascade.

Comparisons to estimates from other population-based cascades are challenged by the heterogeneity and limitations of data sources available across jurisdictions, as well as the varied approaches used to censor for out-migration and death. Cascade indicators calculated with greater uniformity/certainty may be more useful for comparing between studies, such as the percent of VL tests in a given year that are virally suppressed. Our 2010 estimate for the percent of VL tests that were suppressed (79%) was higher than the US 19-jurisdiction study (69% in 2010) [21] and New York City (~72% in 2010) [16] but similar to King County (79% in 2011) [17] and Denmark (80% in 2010) [19]. However, even these comparisons may be limited by the different VL thresholds used to define viral suppression. Regardless, a potentially more valid and informative use of cascade data is to focus on intra-jurisdiction comparisons, such as analyses by time, age, sex and other sociodemographic characteristics. Future analyses of our cohort will attempt to stratify cascade measures by region and Ontario’s priority populations (e.g. gay, bisexual and other men who have sex with men, including trans men; African, Caribbean and Black communities; Indigenous persons; people who use drugs; at-risk women, including trans women), where possible, in order to identify further opportunities for cascade improvement.

Perhaps the most relevant comparison is to the cascade in BC (given the similar cohort-based methodology and Canadian setting), but this is particularly challenged by the different measurement approaches. The more stringent approach used in BC means that the province is often referenced as having lower cascade estimates compared to others [15], a result that would seem at odds with the province’s universal access to ART and progressive HIV policies and programming. In sensitivity analyses—where authors of the BC study adopt indicator definitions more similar to ours and others—the percent of diagnosed PLWH who were suppressed in 2010 increased from 49% to 70% [18], putting it on the higher end of cascade estimates compared to other jurisdictions. These sensitivity analyses further highlight the challenge in comparing between studies and the importance of a standardized approach.

We altered our main indicator definitions in order to calculate upper/lower bounds and found estimates to be particularly sensitive to the more conservative definitions. Alternative definitions/assumptions were based on the range of definitions used in the literature and the nature of our data source. Use of our more conservative “on ART” and “virally suppressed” definitions in the numerator (in which individuals must be on ART or suppressed on all VL tests in a year vs. the most recent test) lowered estimates among diagnosed PLWH by 5–9% and 6–8% absolute percentage points, respectively. In addition, use of our alternative “Upper” diagnosed PLWH definition in the denominator (in which non-nominal HIV-positive diagnoses were included and the LTFU criteria extended to 3 years) decreased these estimates by a further 6–12% and 6–9%, respectively. While these differences are not insignificant, an alternative approach to measuring the number of diagnosed PLWH in New York City led to a 23% absolute change in the percent virally suppressed [56]. The use of these alternative definitions/assumptions demonstrates how indicator estimates can be impacted by both definitions and methodological assumptions, and the importance of presenting a range of plausible estimates instead of what is likely an artificially precise single estimate [14].

Measuring progress towards the UNAIDS 90-90-90 target is a priority for cascade analyses. According to our 2015 estimates, roughly 81% of diagnosed PLWH were on ART (2^nd^ UNAIDS 90 target) and 94% of PLWH on ART were virally suppressed (3^rd^ UNAIDS 90 target). Assuming the national Canadian estimate for the 1^st^ UNAIDS 90 target (80%) [57] applies to Ontario, then approximately 61% of all PLWH in the province would have been virally suppressed in 2015, which is short of the UNAIDS target of 73% when all 90-90-90 targets are simultaneously met. Modeling efforts are underway to estimate the total number of PLWH in Ontario in order to better measure progress towards the UNAIDS 90-90-90 target.

The main strengths of our study were the adoption of methodological recommendations from several reviews and studies. These recommendations include the use of population-based data sources to maximize representativeness of our jurisdiction [10,15], individual-level linkage from diagnosis to suppression to ensure “denominator-denominator” and “numerator-denominator” linkage and to maximize the internal consistency of our outcomes [10], use of a cohort-based approach to facilitate analysis of trends over time and measures of delay (i.e. time to care and suppression) [10,11], use of standardized cascade elements and definitions to improve comparability to other jurisdictions [15], and use of alternative definitions/assumptions to create a range of plausible bounds around main estimates [14].

Our study had limitations related to the analyses. ART status was based on the recording of this information on VL test requisition forms—data that was missing on 17–20% of requisitions. However, we made conservative assumptions on ART status for requisitions with missing data. Further, we relied solely on VL tests as a proxy for care visits and not VL and CD4 tests as done in most studies (CD4 laboratory testing is not centralized in Ontario) [15]. In one cascade analysis, the percent of diagnosed PLWH who were in care was 4% absolute percentage points higher when both CD4 and/or VL tests were used as opposed to VL tests alone [21]. Information on HIV exposure category and race/ethnicity were missing for a large proportion of cohort participants, making it difficult to stratify indicators by these characteristics. Of note, our study team is currently evaluating multiple imputation approaches to address missing sociodemographic and ART data. Although our data were linked at the individual-level and longitudinal in nature, our analyses were mostly cross-sectional by year and based on a linear conceptualization of the HIV cascade (as this is the traditional approach to measuring the cascade). Cross-sectional approaches fail to account for changes in a population over time and do not reflect the dynamic nature of HIV care [10,14]. Unlike many other cascade studies, we did not estimate engagement in “continuous care”, a cross-sectional measure typically defined as two care visits ≥3 months apart in a given year [16,17,21,51]. We were concerned about the potential impact of changing VL testing practices on the validity of this measure. For example, clinicians may recommend less frequent visits/measurement of VL for individuals who are healthy and durably suppressed, with the risk that such individuals could be misclassified as not engaged in continuous care if assessed by frequency of VL testing. A recent clinical cohort study found that 10% of participants not defined as being engaged in continuous care were virally suppressed, yet these individuals would be excluded from the numerator of subsequent cascade steps [58]. Future analyses of our cascade will explore longitudinal approaches informed by non-linear cascade frameworks and measure transitions in and out of different stages [59–62]. Finally, all our analyses were descriptive and future research will explore regression analyses to identify predictors of cascade engagement (pending strategies to address missing data).

There were also limitations related to the nature of our cohort. While we believe our cohort captures the vast majority of diagnosed PLWH in the province, a small number of individuals may have been missed due to the exclusion of non-nominal diagnoses and our indirect censorship for out-migration and death. However, we assume that the majority of non-nominally diagnosed individuals will present for HIV care and be included in the cohort as an individual with a VL test only (no linked diagnostic test). Of note, those diagnosed non-nominally who never connect to care are not included in our cohort, while non-nominally diagnosed individuals are less likely to be included in the newly diagnosed sample (as this sample excludes individuals with a VL test only). Further, as a result of indirectly censoring for death and out-migration (>2 years with no VL test and no VL test in later years), it is likely that some diagnosed individuals were inappropriately removed from our cohort. Indirect censorship is common in other studies of diagnosed PLWH but there is no standardized approach (LTFU rules range from 1.5 to 7 years with no healthcare use) [16,18,35]. Finally, our retrospective LTFU rule may have biased estimates in more current years, as those recently LTFU may not have had time to return to care and be retrospectively included as diagnosed PLWH. Linkage of our cohort to other administrative health care databases is planned in order to strengthen our data source.

In conclusion, our population-based assessment of HIV cascade indicators in Ontario demonstrates substantial improvement from 2000 to 2015. Our results also indicate room for further improvement, particularly among younger individuals. The results in this paper are the first to emerge from our newly created data source, the Ontario HIV Laboratory Cohort, and fill an important gap in our understanding of Ontario’s cascade. Notable limitations inherent to our data source include the exclusion of non-nominal diagnoses; missing ART, CD4 and sociodemographic information; and indirect censorship for out-migration and death. Future efforts will build upon these analyses by exploring non-linear cascade frameworks, stratifying estimates by region and priority population, and strengthening our data source via linkage to additional administrative health care databases.

## Supporting information

S1 Table Number of HIV-positive diagnostic tests by type of identifier (nominal and non-nominal), Ontario, 2000 to 2015. (DOCX)

S2 Table Temporal changes in sex and age distribution among Ontario HIV Laboratory Cohort participants, 2000 to 2015. (DOCX)

S3 Table Comparison of characteristics for HIV-positive nominal diagnostic tests vs. non-nominal tests. (DOCX)

S1 Supporting Information Underlying data for all figures in manuscript. (DOCX)

S2 Supporting Information Flow diagram for creation of newly diagnosed sample from the Public Health Ontario HIV Laboratory datamart and annual number of newly diagnosed individuals from 2000 to 2015. (DOCX)

S3 Supporting Information Cascade indicator estimates by sex and age (for indicators/figures not included in manuscript). (DOCX)
