# Supplementary material for: Trends in HIV care cascade engagement among diagnosed people living with HIV in Ontario, Canada: A retrospective, population-based cohort study
Source: PLoS One. 2019 Jan 4;14(1):e0210096. doi: 10.1371/journal.pone.0210096 (PMC6319701; doi:10.1371/journal.pone.0210096)
Supplement: S1 Table — (DOCX) [file pone.0210096.s001.docx]

**Table A.** Number of HIV-positive diagnostic tests by type of identifier (nominal and non-nominal) and percent non-nominal, Public Health Ontario Laboratory HIV datamart, 2000-2015

| Year | Nominal | Non-nominal | % non-nominal |
| --- | --- | --- | --- |
| 2000 | 476 | 455 | 48.9% |
| 2001 | 545 | 440 | 44.7% |
| 2002 | 701 | 457 | 39.5% |
| 2003 | 722 | 364 | 33.5% |
| 2004 | 793 | 361 | 31.3% |
| 2005 | 784 | 329 | 29.6% |
| 2006 | 863 | 262 | 23.3% |
| 2007 | 783 | 240 | 23.5% |
| 2008 | 837 | 252 | 23.1% |
| 2009 | 754 | 218 | 22.4% |
| 2010 | 775 | 243 | 23.9% |
| 2011 | 788 | 221 | 21.9% |
| 2012 | 677 | 248 | 26.8% |
| 2013 | 625 | 198 | 24.1% |
| 2014 | 724 | 136 | 15.8% |
| 2015 | 722 | 127 | 15.0% |

Data provided by the Public Health Ontario Laboratory
