# Supplementary material for: Trends in HIV care cascade engagement among diagnosed people living with HIV in Ontario, Canada: A retrospective, population-based cohort study
Source: PLoS One. 2019 Jan 4;14(1):e0210096. doi: 10.1371/journal.pone.0210096 (PMC6319701; doi:10.1371/journal.pone.0210096)
Supplement: S2 Table — (DOCX) [file pone.0210096.s002.docx]

**Table A.** Number and percent of cohort participants by sex, Ontario HIV Laboratory Cohort, 2000-2015

| Year | Male | Female | % Male | % Female |
| --- | --- | --- | --- | --- |
| 2000 | 7511 | 1328 | 85.0% | 15.0% |
| 2001 | 7877 | 1485 | 84.1% | 15.9% |
| 2002 | 8295 | 1646 | 83.4% | 16.6% |
| 2003 | 8715 | 1854 | 82.5% | 17.5% |
| 2004 | 9115 | 2003 | 82.0% | 18.0% |
| 2005 | 9504 | 2155 | 81.5% | 18.5% |
| 2006 | 9940 | 2367 | 80.8% | 19.2% |
| 2007 | 10293 | 2529 | 80.3% | 19.7% |
| 2008 | 10687 | 2680 | 80.0% | 20.0% |
| 2009 | 11010 | 2796 | 79.7% | 20.3% |
| 2010 | 11302 | 2831 | 80.0% | 20.0% |
| 2011 | 11634 | 2931 | 79.9% | 20.1% |
| 2012 | 11833 | 3011 | 79.7% | 20.3% |
| 2013 | 12112 | 3089 | 79.7% | 20.3% |
| 2014 | 12406 | 3146 | 79.8% | 20.2% |
| 2015 | 12724 | 3257 | 79.6% | 20.4% |

Data provided by the Public Health Ontario Laboratory

**Table B**. Number and percent of cohort participants by age category, Ontario HIV Laboratory Cohort, 2000-2015

| Year | < 25 | 25-34 | 35-44 | 45-54 | 55-64 | ≥ 65 | < 25 | 25-34 | 35-44 | 45-54 | 55-64 | ≥ 65 |
| --- | --- | --- | --- | --- | --- | --- | --- | --- | --- | --- | --- | --- |
| 2000 | 317 | 1910 | 4005 | 1899 | 519 | 143 | 3.6% | 21.7% | 45.5% | 21.6% | 5.9% | 1.6% |
| 2001 | 343 | 1811 | 4297 | 2083 | 617 | 168 | 3.7% | 19.4% | 46.1% | 22.4% | 6.6% | 1.8% |
| 2002 | 356 | 1784 | 4546 | 2307 | 708 | 207 | 3.6% | 18.0% | 45.9% | 23.3% | 7.1% | 2.1% |
| 2003 | 370 | 1771 | 4780 | 2574 | 825 | 225 | 3.5% | 16.8% | 45.3% | 24.4% | 7.8% | 2.1% |
| 2004 | 386 | 1776 | 4833 | 2898 | 951 | 257 | 3.5% | 16.0% | 43.5% | 26.1% | 8.6% | 2.3% |
| 2005 | 411 | 1753 | 4914 | 3212 | 1088 | 278 | 3.5% | 15.0% | 42.2% | 27.6% | 9.3% | 2.4% |
| 2006 | 429 | 1832 | 4924 | 3582 | 1213 | 325 | 3.5% | 14.9% | 40.0% | 29.1% | 9.9% | 2.6% |
| 2007 | 429 | 1826 | 4811 | 4006 | 1373 | 377 | 3.3% | 14.2% | 37.5% | 31.2% | 10.7% | 2.9% |
| 2008 | 445 | 1867 | 4639 | 4432 | 1537 | 446 | 3.3% | 14.0% | 34.7% | 33.2% | 11.5% | 3.3% |
| 2009 | 450 | 1872 | 4408 | 4865 | 1704 | 511 | 3.3% | 13.6% | 31.9% | 35.2% | 12.3% | 3.7% |
| 2010 | 445 | 1889 | 4178 | 5221 | 1922 | 585 | 3.1% | 13.3% | 29.3% | 36.7% | 13.5% | 4.1% |
| 2011 | 467 | 1905 | 4001 | 5477 | 2119 | 703 | 3.2% | 13.0% | 27.3% | 37.3% | 14.4% | 4.8% |
| 2012 | 460 | 1912 | 3866 | 5600 | 2329 | 792 | 3.1% | 12.8% | 25.8% | 37.4% | 15.6% | 5.3% |
| 2013 | 441 | 1942 | 3716 | 5727 | 2584 | 896 | 2.9% | 12.7% | 24.3% | 37.4% | 16.9% | 5.9% |
| 2014 | 438 | 1983 | 3596 | 5779 | 2875 | 986 | 2.8% | 12.7% | 23.0% | 36.9% | 18.4% | 6.3% |
| 2015 | 469 | 2009 | 3529 | 5737 | 3198 | 1131 | 2.9% | 12.5% | 22.0% | 35.7% | 19.9% | 7.0% |

Data provided by the Public Health Ontario Laboratory
