# Supplementary material for: Trends in HIV care cascade engagement among diagnosed people living with HIV in Ontario, Canada: A retrospective, population-based cohort study
Source: PLoS One. 2019 Jan 4;14(1):e0210096. doi: 10.1371/journal.pone.0210096 (PMC6319701; doi:10.1371/journal.pone.0210096)
Supplement: S3 Table — (DOCX) [file pone.0210096.s003.docx]

**Table A. Comparison of characteristics for nominal HIV-positive diagnostic tests vs. non-nominal tests (1996-2015).** Nominal and non-nominal columns may include same individuals, as some people diagnosed non-nominally received a nominal diagnosis when entering care. LEP form was introduced in 1999 and race/ethnicity collected on this form since 2009. * *p* < 0.0001, ** *p* <0.01, *** *p* <0.05. MSM=men who have sex with men. PWID=people who use injection drugs. LEP=laboratory enhancement program. LTFU=lost to follow up.

| Characteristic | Nominal (N=13,626) | | Non-nominal (N=6,882) | |
| --- | --- | --- | --- | --- |
| Sex (where known) |  |  |  |  |
| Female | 3,516 | 26.1% | 919 | 13.8%^*^ |
| Male | 9,964 | 73.9% | 5,726 | 86.2% |
| Age (where known) |  |  |  |  |
| <25 | 1,421 | 10.5% | 651 | 9.9%^*^ |
| 25-34 | 4,200 | 31.1% | 2,475 | 37.5% |
| 35-44 | 4,482 | 33.2% | 2,283 | 34.6% |
| 45-54 | 2,377 | 17.6% | 897 | 13.6% |
| 55+ | 1,033 | 7.6% | 301 | 4.6% |
| Period of diagnosis |  |  |  |  |
| Prior to 1996 | -- | -- | -- | -- |
| 1996-2000 | 2,533 | 18.6% | 2,786 | 40.5%^*^ |
| 2001-2005 | 3,545 | 26.0% | 1,951 | 28.3% |
| 2006-2010 | 4,012 | 29.4% | 1,215 | 17.7% |
| 2011-2015 | 3,536 | 26.0% | 930 | 13.5% |
| Race/ethnicity (where known) |  |  |  |  |
| White | 1,513 | 51.1% | 529 | 57.6%^*^ |
| Black | 819 | 27.6% | 129 | 14.0% |
| Latin American | 181 | 6.1% | 98 | 10.7% |
| East/Southeast Asian | 158 | 5.3% | 67 | 7.3% |
| South Asian | 118 | 4.0% | 32 | 3.5% |
| Indigenous | 82 | 2.8% | 12 | 1.3% |
| Arab/West Asian | 51 | 1.7% | 18 | 2.0% |
| Other/mixed | 41 | 1.4% | 34 | 3.7% |
| HIV exposure category (where known) |  |  |  |  |
| MSM | 4,068 | 40.8% | 3,838 | 70.9%^*^ |
| Heterosexual | 1,934 | 19.4% | 641 | 11.8%^*^ |
| HIV-endemic | 1,925 | 19.3% | 378 | 7.0%^*^ |
| PWID | 1,187 | 11.9% | 441 | 8.1%^*^ |
| Missing information |  |  |  |  |
| Sex | 146 | 1.1% | 237 | 3.4%^*^ |
| Age | 113 | 0.8% | 275 | 4.0%^*^ |
| Race/ethnicity | 2,102 | 41.5% | 472 | 33.9%^*^ |
| Exposure category | 3,647 | 26.8% | 1,466 | 21.3%^*^ |
| LEP form | 4,770 | 39.5% | 1,897 | 37.1%^**^ |
| Health insurance plan number | 11,045 | 81.1% | -- | -- |
| Region of residence | 268 | 2.0% | 98 | 1.4%^**^ |

Data provided by the Public Health Ontario Laboratory.
