# Supplementary material for: Trends in HIV care cascade engagement among diagnosed people living with HIV in Ontario, Canada: A retrospective, population-based cohort study
Source: PLoS One. 2019 Jan 4;14(1):e0210096. doi: 10.1371/journal.pone.0210096 (PMC6319701; doi:10.1371/journal.pone.0210096)
Supplement: S1 Supporting Information — (DOCX) [file pone.0210096.s004.docx]

1. **Main Indicator Estimates (Figure 2)**

**Table A.** Number of people with diagnosed HIV living in Ontario, 2000 to 2015

| **Year** | **Main estimate** | **Upper estimate** |
| --- | --- | --- |
|  | Nominal HIV-positive diagnostic test **and/or** HIV viral load test*, **and not** administratively lost to follow-up after two years | Nominal or non-nominal HIV-positive diagnostic test **and/or** HIV viral load test*, **and not** administratively lost to follow-up after three years |
| **2000** | 8,859 | 11,389 |
| **2001** | 9,367 | 11,787 |
| **2002** | 9,945 | 12,317 |
| **2003** | 10,574 | 12,742 |
| **2004** | 11,125 | 13,219 |
| **2005** | 11,672 | 13,648 |
| **2006** | 12,318 | 14,104 |
| **2007** | 12,832 | 14,477 |
| **2008** | 13,377 | 14,951 |
| **2009** | 13,819 | 15,298 |
| **2010** | 14,255 | 15,712 |
| **2011** | 14,699 | 16,162 |
| **2012** | 14,999 | 16,527 |
| **2013** | 15,346 | 16,764 |
| **2014** | 15,694 | 17,035 |
| **2015** | 16,110 | 17,423 |

**Notes:** Data provided by the Public Health Ontario Laboratory. See manuscript for more information on indicator definitions. * Individuals with no nominal HIV-positive diagnostic test, and all undetectable VL tests, were not included if there was evidence of being HIV-negative.

**Table B.** Number and percent of people with diagnosed HIV living in Ontario who were in care, 2000 to 2015

| **Year** | **Main Estimate** | | | **Lower Estimate** | | |
| --- | --- | --- | --- | --- | --- | --- |
|  | In care (Numerator) | Diagnosed (Denominator) | % | In care (Numerator) | Diagnosed (Denominator) | % |
|  | At least one VL | Diagnosed “Main” Estimate |  | At least one VL | Diagnosed “Upper” Estimate |  |
| **2000** | 7,203 | 8,859 | 81.3% | 7,203 | 11,389 | 63.2% |
| **2001** | 7,669 | 9,367 | 81.9% | 7,669 | 11,787 | 65.1% |
| **2002** | 8,232 | 9,945 | 82.8% | 8,232 | 12,317 | 66.8% |
| **2003** | 8,775 | 10,574 | 83.0% | 8,775 | 12,742 | 68.9% |
| **2004** | 9,336 | 11,125 | 83.9% | 9,336 | 13,219 | 70.6% |
| **2005** | 9,848 | 11,672 | 84.4% | 9,848 | 13,648 | 72.2% |
| **2006** | 10,402 | 12,318 | 84.4% | 10,402 | 14,104 | 73.8% |
| **2007** | 10,814 | 12,832 | 84.3% | 10,814 | 14,477 | 74.7% |
| **2008** | 11,317 | 13,377 | 84.6% | 11,317 | 14,951 | 75.7% |
| **2009** | 11,721 | 13,819 | 84.8% | 11,721 | 15,298 | 76.6% |
| **2010** | 12,094 | 14,255 | 84.8% | 12,094 | 15,712 | 77.0% |
| **2011** | 12,581 | 14,699 | 85.6% | 12,581 | 16,162 | 77.8% |
| **2012** | 12,883 | 14,999 | 85.9% | 12,883 | 16,527 | 78.0% |
| **2013** | 13,246 | 15,346 | 86.3% | 13,246 | 16,764 | 79.0% |
| **2014** | 13,635 | 15,694 | 86.9% | 13,635 | 17,035 | 80.0% |
| **2015** | 14,065 | 16,110 | 87.3% | 14,065 | 17,423 | 80.7% |

**Notes:** Data provided by the Public Health Ontario Laboratory. See manuscript for more details on indicator definitions.

**Table C.** Number and percent of people with diagnosed HIV living in Ontario who were on ART, 2000 to 2015

| **Year** | **Main Estimate** | | | **Upper Estimate** | | | **Lower Estimate** | | |
| --- | --- | --- | --- | --- | --- | --- | --- | --- | --- |
|  | On ART  (Numerator) | Diagnosed (Denominator) | % | On ART  (Numerator) | Diagnosed (Denominator) | % | On ART  (Numerator) | Diagnosed (Denominator) | % |
|  | Documented on ART, or ART status missing and suppressed, on **last** VL test | Diagnosed “Main” Estimate |  | Documented on ART, or ART status missing and suppressed, on **any** VL test | Diagnosed “Main” Estimate |  | Documented on ART, or ART status missing and suppressed, on **all** VL tests | Diagnosed “Upper” Estimate |  |
| **2000** | 4,880 | 8,859 | 55.1% | 5,335 | 8,859 | 60.2% | 3,909 | 11,389 | 34.3% |
| **2001** | 5,178 | 9,367 | 55.3% | 5,585 | 9,367 | 59.6% | 4,183 | 11,787 | 35.5% |
| **2002** | 5,555 | 9,945 | 55.9% | 6,012 | 9,945 | 60.5% | 4,556 | 12,317 | 37.0% |
| **2003** | 5,932 | 10,574 | 56.1% | 6,333 | 10,574 | 59.9% | 4,914 | 12,742 | 38.6% |
| **2004** | 6,385 | 11,125 | 57.4% | 6,735 | 11,125 | 60.5% | 5,376 | 13,219 | 40.7% |
| **2005** | 6,861 | 11,672 | 58.8% | 7,213 | 11,672 | 61.8% | 5,853 | 13,648 | 42.9% |
| **2006** | 7,432 | 12,318 | 60.3% | 7,723 | 12,318 | 62.7% | 6,446 | 14,104 | 45.7% |
| **2007** | 8,003 | 12,832 | 62.4% | 8,294 | 12,832 | 64.6% | 6,997 | 14,477 | 48.3% |
| **2008** | 8,769 | 13,377 | 65.6% | 9,006 | 13,377 | 67.3% | 7,712 | 14,951 | 51.6% |
| **2009** | 9,397 | 13,819 | 68.0% | 9,618 | 13,819 | 69.6% | 8,276 | 15,298 | 54.1% |
| **2010** | 9,994 | 14,255 | 70.1% | 10,220 | 14,255 | 71.7% | 8,955 | 15,712 | 57.0% |
| **2011** | 10,685 | 14,699 | 72.7% | 10,864 | 14,699 | 73.9% | 9,699 | 16,162 | 60.0% |
| **2012** | 11,310 | 14,999 | 75.4% | 11,489 | 14,999 | 76.6% | 10,350 | 16,527 | 62.6% |
| **2013** | 11,920 | 15,346 | 77.7% | 12,064 | 15,346 | 78.6% | 10,970 | 16,764 | 65.4% |
| **2014** | 12,447 | 15,694 | 79.3% | 12,602 | 15,694 | 80.3% | 11,584 | 17,035 | 68.0% |
| **2015** | 13,059 | 16,110 | 81.1% | 13,212 | 16,110 | 82.0% | 12,181 | 17,423 | 69.9% |

**Notes:** Data provided by the Public Health Ontario Laboratory. See manuscript for more details on indicator definitions.

**Table D.** Number and percent of people with diagnosed HIV living in Ontario who are virally suppressed, 2000 to 2015

| **Year** | **Main Estimate** | | | **Upper Estimate** | | | **Lower Estimate** | | |
| --- | --- | --- | --- | --- | --- | --- | --- | --- | --- |
|  | VL suppressed  (Numerator) | Diagnosed (Denominator) | % | VL suppressed  (Numerator) | Diagnosed (Denominator) | % | VL suppressed  (Numerator) | Diagnosed (Denominator) | % |
|  | VL less than 200 copies per ml on **last** VL test | Diagnosed “Main” Estimate |  | VL less than 200 copies per ml on **any** VL test | Diagnosed “Main” Estimate |  | VL less than 200 copies per ml on **all** VL tests | Diagnosed “Upper” Estimate |  |
| **2000** | 3,607 | 8,859 | 40.7% | 4,087 | 8,859 | 46.1% | 2,655 | 11,389 | 23.3% |
| **2001** | 4,029 | 9,367 | 43.0% | 4,537 | 9,367 | 48.4% | 3,081 | 11,787 | 26.1% |
| **2002** | 4,464 | 9,945 | 44.9% | 4,966 | 9,945 | 49.9% | 3,516 | 12,317 | 28.5% |
| **2003** | 4,966 | 10,574 | 47.0% | 5,432 | 10,574 | 51.4% | 3,944 | 12,742 | 31.0% |
| **2004** | 5,544 | 11,125 | 49.8% | 5,955 | 11,125 | 53.5% | 4,480 | 13,219 | 33.9% |
| **2005** | 6,086 | 11,672 | 52.1% | 6,514 | 11,672 | 55.8% | 5,053 | 13,648 | 37.0% |
| **2006** | 6,824 | 12,318 | 55.4% | 7,201 | 12,318 | 58.5% | 5,712 | 14,104 | 40.5% |
| **2007** | 7,518 | 12,832 | 58.6% | 7,883 | 12,832 | 61.4% | 6,363 | 14,477 | 44.0% |
| **2008** | 8,349 | 13,377 | 62.4% | 8,671 | 13,377 | 64.8% | 7,130 | 14,951 | 47.7% |
| **2009** | 9,024 | 13,819 | 65.3% | 9,327 | 13,819 | 67.5% | 7,823 | 15,298 | 51.1% |
| **2010** | 9,606 | 14,255 | 67.4% | 9,946 | 14,255 | 69.8% | 8,452 | 15,712 | 53.8% |
| **2011** | 10,328 | 14,699 | 70.3% | 10,580 | 14,699 | 72.0% | 9,189 | 16,162 | 56.9% |
| **2012** | 10,952 | 14,999 | 73.0% | 11,202 | 14,999 | 74.7% | 9,849 | 16,527 | 59.6% |
| **2013** | 11,574 | 15,346 | 75.4% | 11,807 | 15,346 | 76.9% | 10,502 | 16,764 | 62.6% |
| **2014** | 12,136 | 15,694 | 77.3% | 12,364 | 15,694 | 78.8% | 11,100 | 17,035 | 65.2% |
| **2015** | 12,809 | 16,110 | 79.5% | 13,016 | 16,110 | 80.8% | 11,742 | 17,423 | 67.4% |

**Notes:** Data provided by the Public Health Ontario Laboratory. See manuscript for more details on indicator definitions.

1. **Conditional estimates (Figure 3)**

**Table E.** Number and percent of people with diagnosed HIV living in Ontario in care who were on ART, 2000 to 2015

| **Year** | **Main Estimate** | | | **Upper Estimate** | | | **Lower Estimate** | | |
| --- | --- | --- | --- | --- | --- | --- | --- | --- | --- |
|  | On ART  (Numerator) | In care (Denominator) | % | On ART (Numerator) | In care  (Denominator) | % | On ART  Numerator) | In care (Denominator) | % |
|  | Documented on ART, or ART status missing and suppressed, on **last** VL test | At least one VL |  | Documented on ART, or ART status missing and suppressed, on **any** VL test | At least one VL |  | Documented on ART, or ART status missing and suppressed, on **all** VL tests | At least one VL |  |
| **2000** | 4,880 | 7,203 | 67.7% | 5,335 | 7,203 | 74.1% | 3,909 | 7,203 | 54.3% |
| **2001** | 5,178 | 7,669 | 67.5% | 5,585 | 7,669 | 72.8% | 4,183 | 7,669 | 54.5% |
| **2002** | 5,555 | 8,232 | 67.5% | 6,012 | 8,232 | 73.0% | 4,556 | 8,232 | 55.3% |
| **2003** | 5,932 | 8,775 | 67.6% | 6,333 | 8,775 | 72.2% | 4,914 | 8,775 | 56.0% |
| **2004** | 6,385 | 9,336 | 68.4% | 6,735 | 9,336 | 72.1% | 5,376 | 9,336 | 57.6% |
| **2005** | 6,861 | 9,848 | 69.7% | 7,213 | 9,848 | 73.2% | 5,853 | 9,848 | 59.4% |
| **2006** | 7,432 | 10,402 | 71.4% | 7,723 | 10,402 | 74.2% | 6,446 | 10,402 | 62.0% |
| **2007** | 8,003 | 10,814 | 74.0% | 8,294 | 10,814 | 76.7% | 6,997 | 10,814 | 64.7% |
| **2008** | 8,769 | 11,317 | 77.5% | 9,006 | 11,317 | 79.6% | 7,712 | 11,317 | 68.1% |
| **2009** | 9,397 | 11,721 | 80.2% | 9,618 | 11,721 | 82.1% | 8,276 | 11,721 | 70.6% |
| **2010** | 9,994 | 12,094 | 82.6% | 10,220 | 12,094 | 84.5% | 8,955 | 12,094 | 74.0% |
| **2011** | 10,685 | 12,581 | 84.9% | 10,864 | 12,581 | 86.4% | 9,699 | 12,581 | 77.1% |
| **2012** | 11,310 | 12,883 | 87.8% | 11,489 | 12,883 | 89.2% | 10,350 | 12,883 | 80.3% |
| **2013** | 11,920 | 13,246 | 90.0% | 12,064 | 13,246 | 91.1% | 10,970 | 13,246 | 82.8% |
| **2014** | 12,447 | 13,635 | 91.3% | 12,602 | 13,635 | 92.4% | 11,584 | 13,635 | 85.0% |
| **2015** | 13,059 | 14,065 | 92.8% | 13,212 | 14,065 | 93.9% | 12,181 | 14,065 | 86.6% |

**Notes:** Data provided by the Public Health Ontario Laboratory. See manuscript for more details on indicator definitions.

**Table F.** Number and percent of people with diagnosed HIV living in Ontario in care who were virally suppressed, 2000 to 2015

| **Year** | **Main Estimate** | | | **Upper Estimate** | | | **Lower Estimate** | | |
| --- | --- | --- | --- | --- | --- | --- | --- | --- | --- |
|  | VL suppressed  (Numerator) | In care  (Denominator) | % | VL suppressed  (Numerator) | In care  (Denominator) | % | VL suppressed  (Numerator) | In care  (Denominator) | % |
|  | VL less than 200 copies per ml on **last** VL test | At least one VL |  | VL less than 200 copies per ml on **any** VL test | At least one VL |  | VL less than 200 copies per ml on **all** VL tests | At least one VL |  |
| **2000** | 3,607 | 7,203 | 50.1% | 4,087 | 7,203 | 56.7% | 2,655 | 7,203 | 36.9% |
| **2001** | 4,029 | 7,669 | 52.5% | 4,537 | 7,669 | 59.2% | 3,081 | 7,669 | 40.2% |
| **2002** | 4,464 | 8,232 | 54.2% | 4,966 | 8,232 | 60.3% | 3,516 | 8,232 | 42.7% |
| **2003** | 4,966 | 8,775 | 56.6% | 5,432 | 8,775 | 61.9% | 3,944 | 8,775 | 44.9% |
| **2004** | 5,544 | 9,336 | 59.4% | 5,955 | 9,336 | 63.8% | 4,480 | 9,336 | 48.0% |
| **2005** | 6,086 | 9,848 | 61.8% | 6,514 | 9,848 | 66.1% | 5,053 | 9,848 | 51.3% |
| **2006** | 6,824 | 10,402 | 65.6% | 7,201 | 10,402 | 69.2% | 5,712 | 10,402 | 54.9% |
| **2007** | 7,518 | 10,814 | 69.5% | 7,883 | 10,814 | 72.9% | 6,363 | 10,814 | 58.8% |
| **2008** | 8,349 | 11,317 | 73.8% | 8,671 | 11,317 | 76.6% | 7,130 | 11,317 | 63.0% |
| **2009** | 9,024 | 11,721 | 77.0% | 9,327 | 11,721 | 79.6% | 7,823 | 11,721 | 66.7% |
| **2010** | 9,606 | 12,094 | 79.4% | 9,946 | 12,094 | 82.2% | 8,452 | 12,094 | 69.9% |
| **2011** | 10,328 | 12,581 | 82.1% | 10,580 | 12,581 | 84.1% | 9,189 | 12,581 | 73.0% |
| **2012** | 10,952 | 12,883 | 85.0% | 11,202 | 12,883 | 87.0% | 9,849 | 12,883 | 76.4% |
| **2013** | 11,574 | 13,246 | 87.4% | 11,807 | 13,246 | 89.1% | 10,502 | 13,246 | 79.3% |
| **2014** | 12,136 | 13,635 | 89.0% | 12,364 | 13,635 | 90.7% | 11,100 | 13,635 | 81.4% |
| **2015** | 12,809 | 14,065 | 91.1% | 13,016 | 14,065 | 92.5% | 11,742 | 14,065 | 83.5% |

**Notes:** Data provided by the Public Health Ontario Laboratory. See manuscript for more details on indicator definitions.

**Table G.** Number and percent of people with diagnosed HIV living in Ontario on ART who were virally suppressed, 2000 to 2015

| **Year** | **Main Estimate** | | | **Upper Estimate** | | | **Lower Estimate** | | |
| --- | --- | --- | --- | --- | --- | --- | --- | --- | --- |
|  | VL suppressed  (Numerator) | On ART (Denominator) | % | VL suppressed  (Numerator) | On ART (Denominator) | % | VL suppressed  (Numerator) | On ART (Denominator) | % |
|  | VL less than 200 copies per ml, and known on ART or ART status missing, on **last** VL test | Known on ART, or ART status missing, on **last** VL test |  | VL less than 200 copies per ml, and known on ART or ART status missing, on **any** VL test | Known on ART, or ART status missing, on **any** VL test |  | VL less than 200 copies per ml, and known on ART or ART status missing, on **all** VL tests | Known on ART, or ART status missing, on **all** VL tests |  |
| **2000** | 3,495 | 5,530 | 63.2% | 3,974 | 6,004 | 66.2% | 2,527 | 4,620 | 54.7% |
| **2001** | 3,921 | 5,888 | 66.6% | 4,423 | 6,340 | 69.8% | 2,950 | 4,979 | 59.2% |
| **2002** | 4,340 | 6,310 | 68.8% | 4,855 | 6,857 | 70.8% | 3,385 | 5,351 | 63.3% |
| **2003** | 4,810 | 6,701 | 71.8% | 5,299 | 7,267 | 72.9% | 3,763 | 5,741 | 65.5% |
| **2004** | 5,414 | 7,220 | 75.0% | 5,830 | 7,740 | 75.3% | 4,322 | 6,216 | 69.5% |
| **2005** | 5,928 | 7,663 | 77.4% | 6,362 | 8,184 | 77.7% | 4,879 | 6,650 | 73.4% |
| **2006** | 6,620 | 8,248 | 80.3% | 7,009 | 8,743 | 80.2% | 5,487 | 7,234 | 75.9% |
| **2007** | 7,336 | 8,759 | 83.8% | 7,712 | 9,222 | 83.6% | 6,149 | 7,796 | 78.9% |
| **2008** | 8,170 | 9,500 | 86.0% | 8,517 | 9,953 | 85.6% | 6,925 | 8,535 | 81.1% |
| **2009** | 8,847 | 10,036 | 88.2% | 9,168 | 10,438 | 87.8% | 7,603 | 8,998 | 84.5% |
| **2010** | 9,433 | 10,552 | 89.4% | 9,781 | 10,913 | 89.6% | 8,245 | 9,610 | 85.8% |
| **2011** | 10,149 | 11,204 | 90.6% | 10,417 | 11,516 | 90.5% | 8,981 | 10,297 | 87.2% |
| **2012** | 10,788 | 11,795 | 91.5% | 11,055 | 12,053 | 91.7% | 9,663 | 10,929 | 88.4% |
| **2013** | 11,415 | 12,366 | 92.3% | 11,663 | 12,566 | 92.8% | 10,305 | 11,552 | 89.2% |
| **2014** | 11,992 | 12,821 | 93.5% | 12,233 | 13,010 | 94.0% | 10,912 | 12,103 | 90.2% |
| **2015** | 12,647 | 13,395 | 94.4% | 12,882 | 13,543 | 95.1% | 11,544 | 12,706 | 90.9% |

**Notes:** Data provided by the Public Health Ontario Laboratory. See manuscript for more details on indicator definitions.

1. **Longitudinal cascade estimates (Figure 4)**

**Table H.** Number and percent of people newly diagnosed with HIV in Ontario who were linked to care within a certain period of time after HIV diagnosis, 2000 to 2014

| **Year** | **Main Estimate** | | | | | | | | | | |
| --- | --- | --- | --- | --- | --- | --- | --- | --- | --- | --- | --- |
|  | Newly diagnosed (Denominator) | Linked to care  (Numerator) | | | | | | | | | |
|  | Newly diagnosed “Main” Estimate | Three months or earlier | | Three to six months | | Six to 12 months | | More than 12 months | | No linked VL | |
| **2000** | 368 | 248 | 67.4% | 21 | 5.7% | 10 | 2.7% | 20 | 5.4% | 69 | 18.8% |
| **2001** | 424 | 275 | 64.9% | 35 | 8.3% | 9 | 2.1% | 23 | 5.4% | 82 | 19.3% |
| **2002** | 542 | 384 | 70.8% | 55 | 10.1% | 15 | 2.8% | 20 | 3.7% | 68 | 12.5% |
| **2003** | 553 | 398 | 72.0% | 41 | 7.4% | 18 | 3.3% | 21 | 3.8% | 75 | 13.6% |
| **2004** | 570 | 441 | 77.4% | 31 | 5.4% | 15 | 2.6% | 24 | 4.2% | 59 | 10.4% |
| **2005** | 567 | 418 | 73.7% | 45 | 7.9% | 22 | 3.9% | 23 | 4.1% | 59 | 10.4% |
| **2006** | 599 | 465 | 77.6% | 41 | 6.8% | 19 | 3.2% | 17 | 2.8% | 57 | 9.5% |
| **2007** | 555 | 416 | 75.0% | 40 | 7.2% | 11 | 2.0% | 22 | 4.0% | 66 | 11.9% |
| **2008** | 574 | 407 | 70.9% | 55 | 9.6% | 23 | 4.0% | 16 | 2.8% | 73 | 12.7% |
| **2009** | 523 | 402 | 76.9% | 45 | 8.6% | 13 | 2.5% | 11 | 2.1% | 52 | 9.9% |
| **2010** | 530 | 422 | 79.6% | 42 | 7.9% | 7 | 1.3% | 11 | 2.1% | 48 | 9.1% |
| **2011** | 528 | 425 | 80.5% | 29 | 5.5% | 13 | 2.5% | 13 | 2.5% | 48 | 9.1% |
| **2012** | 450 | 369 | 82.0% | 28 | 6.2% | 12 | 2.7% | 6 | 1.3% | 35 | 7.8% |
| **2013** | 415 | 351 | 84.6% | 15 | 3.6% | 6 | 1.4% | 4 | 1.0% | 39 | 9.4% |
| **2014** | 473 | 387 | 81.8% | 23 | 4.9% | 13 | 2.7% | 2 | 0.4% | 48 | 10.1% |

**Notes:** Data provided by the Public Health Ontario Laboratory. See manuscript for more details on indicator definitions.

**Table I.** Median number of days from diagnosis to linkage to care and interquartile range, Ontario, 2000 to 2014

| Year | Median | Q1 | Q3 |
| --- | --- | --- | --- |
| 2000 | 35 | 22 | 68 |
| 2001 | 40 | 26 | 72 |
| 2002 | 48 | 31 | 74 |
| 2003 | 44 | 28 | 68 |
| 2004 | 41 | 26 | 65 |
| 2005 | 43 | 27 | 71 |
| 2006 | 38 | 25 | 64 |
| 2007 | 38 | 23 | 68 |
| 2008 | 36 | 23 | 70 |
| 2009 | 35 | 21 | 58 |
| 2010 | 34 | 21 | 56 |
| 2011 | 27 | 14 | 54 |
| 2012 | 27 | 15 | 54 |
| 2013 | 21 | 11 | 41 |
| 2014 | 24 | 12 | 43 |

**Notes:** Data provided by the Public Health Ontario Laboratory. See manuscript for more details on indicator definitions.

**Table J.** Number and percent of people newly diagnosed with HIV in Ontario who were virally suppressed within a certain period of time after HIV diagnosis, 2000 to 2013

| **Year** | **Main Estimate** | | | | | | | | | | |
| --- | --- | --- | --- | --- | --- | --- | --- | --- | --- | --- | --- |
|  | Newly diagnosed (Denominator) | Time to viral suppression (VL less than 200 copies per ml)  (Numerator) | | | | | | | | | |
|  | Newly diagnosed “Main” Estimate | Six months or earlier | | Six to 12 months | | More than 12 months | | No suppressed VL | | No linked VL | |
| **2000** | 368 | 81 | 22.0% | 41 | 11.1% | 125 | 34.0% | 52 | 14.1% | 69 | 18.8% |
| **2001** | 424 | 98 | 23.1% | 42 | 9.9% | 137 | 32.3% | 65 | 15.3% | 82 | 19.3% |
| **2002** | 542 | 114 | 21.0% | 83 | 15.3% | 206 | 38.0% | 71 | 13.1% | 68 | 12.5% |
| **2003** | 553 | 114 | 20.6% | 71 | 12.8% | 214 | 38.7% | 79 | 14.3% | 75 | 13.6% |
| **2004** | 570 | 118 | 20.7% | 81 | 14.2% | 255 | 44.7% | 57 | 10.0% | 59 | 10.4% |
| **2005** | 567 | 90 | 15.9% | 75 | 13.2% | 257 | 45.3% | 86 | 15.2% | 59 | 10.4% |
| **2006** | 599 | 109 | 18.2% | 77 | 12.9% | 262 | 43.7% | 94 | 15.7% | 57 | 9.5% |
| **2007** | 555 | 107 | 19.3% | 68 | 12.3% | 234 | 42.2% | 80 | 14.4% | 66 | 11.9% |
| **2008** | 574 | 145 | 25.3% | 68 | 11.8% | 215 | 37.5% | 73 | 12.7% | 73 | 12.7% |
| **2009** | 523 | 134 | 25.6% | 88 | 16.8% | 172 | 32.9% | 77 | 14.7% | 52 | 9.9% |
| **2010** | 530 | 136 | 25.7% | 99 | 18.7% | 157 | 29.6% | 90 | 17.0% | 48 | 9.1% |
| **2011** | 528 | 156 | 29.5% | 107 | 20.3% | 131 | 24.8% | 86 | 16.3% | 48 | 9.1% |
| **2012** | 450 | 170 | 37.8% | 106 | 23.6% | 78 | 17.3% | 61 | 13.6% | 35 | 7.8% |
| **2013** | 415 | 172 | 41.4% | 93 | 22.4% | 55 | 13.3% | 56 | 13.5% | 39 | 9.4% |

**Notes:** Data provided by the Public Health Ontario Laboratory. See manuscript for more details on indicator definitions.

**Table K.** Median number of days from diagnosis to viral suppression and interquartile range, Ontario, 2000 to 2014

| Year | Median | Q1 | Q3 |
| --- | --- | --- | --- |
| 2000 | 402 | 148 | 1498 |
| 2001 | 343 | 145 | 1350 |
| 2002 | 427 | 168 | 1534 |
| 2003 | 457 | 167 | 1647 |
| 2004 | 531 | 178 | 1414 |
| 2005 | 614 | 202 | 1469 |
| 2006 | 554 | 188 | 1350 |
| 2007 | 479 | 175 | 1176 |
| 2008 | 374 | 155 | 958 |
| 2009 | 278 | 147 | 759 |
| 2010 | 272 | 146 | 646 |
| 2011 | 224 | 134 | 510 |
| 2012 | 194 | 119 | 336 |
| 2013 | 172 | 112 | 285 |

**Notes:** Data provided by the Public Health Ontario Laboratory. See manuscript for more details on indicator definitions.

1. **Cascade estimates related to viral suppression by sex and age (Figure 5)**

**Table L.** Number and percent of people with diagnosed HIV living in Ontario who were virally suppressed by sex, 2000 to 2015

| **Year** | **Male** | | | **Female** | | |
| --- | --- | --- | --- | --- | --- | --- |
|  | Virally suppressed  (numerator) | Diagnosed (denominator) | % | Virally suppressed  (numerator) | Diagnosed (denominator) | % |
|  | VL less than 200 copies per ml on last VL test | Nominal HIV-positive diagnostic test and/or ≥1 VL test, and not LTFU after 2 years |  | VL less than 200 copies per ml on last VL test | Nominal HIV-positive diagnostic test and/or ≥1 VL test, and not LTFU after 2 years |  |
| **2000** | 3,125 | 7,511 | 41.6% | 482 | 1,328 | 36.3% |
| **2001** | 3,458 | 7,877 | 43.9% | 571 | 1,485 | 38.5% |
| **2002** | 3,803 | 8,295 | 45.8% | 661 | 1,646 | 40.2% |
| **2003** | 4,192 | 8,715 | 48.1% | 774 | 1,854 | 41.7% |
| **2004** | 4,665 | 9,115 | 51.2% | 878 | 2,003 | 43.8% |
| **2005** | 5,066 | 9,504 | 53.3% | 1,015 | 2,155 | 47.1% |
| **2006** | 5,626 | 9,940 | 56.6% | 1,198 | 2,367 | 50.6% |
| **2007** | 6,163 | 10,293 | 59.9% | 1,354 | 2,529 | 53.5% |
| **2008** | 6,832 | 10,687 | 63.9% | 1,516 | 2,680 | 56.6% |
| **2009** | 7,337 | 11,010 | 66.6% | 1,684 | 2,796 | 60.2% |
| **2010** | 7,761 | 11,302 | 68.7% | 1,760 | 2,831 | 62.2% |
| **2011** | 8,323 | 11,634 | 71.5% | 1,923 | 2,931 | 65.6% |
| **2012** | 8,752 | 11,833 | 74.0% | 2,102 | 3,011 | 69.8% |
| **2013** | 9,300 | 12,112 | 76.8% | 2,193 | 3,089 | 71.0% |
| **2014** | 9,735 | 12,406 | 78.5% | 2,320 | 3,146 | 73.7% |
| **2015** | 10,229 | 12,724 | 80.4% | 2,491 | 3,257 | 76.5% |

**Notes:** Data provided by the Public Health Ontario Laboratory. See manuscript for more details on indicator definitions.

**Table M.** Number and percent of people newly diagnosed with HIV in Ontario who achieved viral suppression within six months of diagnosis, 2000 to 2014

| **Year** | **Male** | | | **Female** | | |
| --- | --- | --- | --- | --- | --- | --- |
|  | Time to viral suppression  (numerator) | Newly diagnosed  (denominator) | % | Time to viral suppression  (numerator) | Newly diagnosed  (denominator) | % |
|  | Suppressed VL within 6 months of HIV diagnosis | Nominal HIV-positive diagnostic test and no evidence of previous diagnosis |  | Suppressed VL within 6 months of HIV diagnosis | Nominal HIV-positive diagnostic test and no evidence of previous diagnosis |  |
| **2000** | 55 | 264 | 20.8% | 26 | 102 | 25.5% |
| **2001** | 59 | 284 | 20.8% | 38 | 138 | 27.5% |
| **2002** | 76 | 361 | 21.1% | 37 | 178 | 20.8% |
| **2003** | 74 | 367 | 20.2% | 40 | 182 | 22.0% |
| **2004** | 80 | 396 | 20.2% | 38 | 172 | 22.1% |
| **2005** | 64 | 419 | 15.3% | 26 | 146 | 17.8% |
| **2006** | 76 | 408 | 18.6% | 33 | 188 | 17.6% |
| **2007** | 80 | 402 | 19.9% | 26 | 150 | 17.3% |
| **2008** | 100 | 416 | 24.0% | 44 | 153 | 28.8% |
| **2009** | 92 | 399 | 23.1% | 42 | 122 | 34.4% |
| **2010** | 102 | 409 | 24.9% | 32 | 114 | 28.1% |
| **2011** | 116 | 401 | 28.9% | 40 | 122 | 32.8% |
| **2012** | 131 | 337 | 38.9% | 39 | 109 | 35.8% |
| **2013** | 143 | 335 | 42.7% | 27 | 76 | 35.5% |
| **2014** | 159 | 371 | 42.9% | 35 | 96 | 36.5% |

**Notes:** Data provided by the Public Health Ontario Laboratory. See manuscript for more details on indicator definitions.

**Table N.** Number and percent of people with diagnosed HIV living in Ontario who were virally suppressed by age category, 2000 to 2015

| Year | ≤24 | | | 25-34 | | | 35-44 | | | 45-54 | | | ≥55 | | |
| --- | --- | --- | --- | --- | --- | --- | --- | --- | --- | --- | --- | --- | --- | --- | --- |
|  | Virally  supp. | Diagnosed | % | Virally  supp. | Diagnosed | % | Virally  supp. | Diagnosed | % | Virally  supp. | Diagnosed | % | Virally  supp. | Diagnosed | % |
| 2000 | 82 | 317 | 25.9% | 635 | 1,910 | 33.2% | 1,695 | 4,005 | 42.3% | 861 | 1,899 | 45.3% | 330 | 662 | 49.8% |
| 2001 | 101 | 343 | 29.4% | 587 | 1,811 | 32.4% | 1,884 | 4,297 | 43.8% | 1,036 | 2,083 | 49.7% | 420 | 785 | 53.5% |
| 2002 | 111 | 356 | 31.2% | 598 | 1,784 | 33.5% | 2,049 | 4,546 | 45.1% | 1,209 | 2,307 | 52.4% | 496 | 915 | 54.2% |
| 2003 | 117 | 370 | 31.6% | 586 | 1,771 | 33.1% | 2,221 | 4,780 | 46.5% | 1,409 | 2,574 | 54.7% | 633 | 1,050 | 60.3% |
| 2004 | 126 | 386 | 32.6% | 604 | 1,776 | 34.0% | 2,384 | 4,833 | 49.3% | 1,674 | 2,898 | 57.8% | 756 | 1,208 | 62.6% |
| 2005 | 120 | 411 | 29.2% | 620 | 1,753 | 35.4% | 2,502 | 4,914 | 50.9% | 1,940 | 3,212 | 60.4% | 903 | 1,366 | 66.1% |
| 2006 | 151 | 429 | 35.2% | 677 | 1,832 | 37.0% | 2,644 | 4,924 | 53.7% | 2,254 | 3,582 | 62.9% | 1,095 | 1,538 | 71.2% |
| 2007 | 158 | 429 | 36.8% | 694 | 1,826 | 38.0% | 2,715 | 4,811 | 56.4% | 2,663 | 4,006 | 66.5% | 1,288 | 1,750 | 73.6% |
| 2008 | 170 | 445 | 38.2% | 762 | 1,867 | 40.8% | 2,776 | 4,639 | 59.8% | 3,105 | 4,432 | 70.1% | 1,535 | 1,983 | 77.4% |
| 2009 | 195 | 450 | 43.3% | 834 | 1,872 | 44.6% | 2,723 | 4,408 | 61.8% | 3,512 | 4,865 | 72.2% | 1,757 | 2,215 | 79.3% |
| 2010 | 221 | 445 | 49.7% | 946 | 1,889 | 50.1% | 2,646 | 4,178 | 63.3% | 3,812 | 5,221 | 73.0% | 1,978 | 2,507 | 78.9% |
| 2011 | 218 | 467 | 46.7% | 1,034 | 1,905 | 54.3% | 2,655 | 4,001 | 66.4% | 4,116 | 5,477 | 75.2% | 2,296 | 2,822 | 81.4% |
| 2012 | 237 | 460 | 51.5% | 1,142 | 1,912 | 59.7% | 2,656 | 3,866 | 68.7% | 4,312 | 5,600 | 77.0% | 2,588 | 3,121 | 82.9% |
| 2013 | 245 | 441 | 55.6% | 1,230 | 1,942 | 63.3% | 2,684 | 3,716 | 72.2% | 4,500 | 5,727 | 78.6% | 2,909 | 3,480 | 83.6% |
| 2014 | 254 | 438 | 58.0% | 1,332 | 1,983 | 67.2% | 2,622 | 3,596 | 72.9% | 4,656 | 5,779 | 80.6% | 3,266 | 3,861 | 84.6% |
| 2015 | 301 | 469 | 64.2% | 1,365 | 2,009 | 67.9% | 2,695 | 3,529 | 76.4% | 4,718 | 5,737 | 82.2% | 3,717 | 4,329 | 85.9% |

**Notes:** Data provided by the Public Health Ontario Laboratory. See manuscript for more details on indicator definitions. Percentages calculated using ‘virally supp.” in the numerator and ‘diagnosed’ in the denominator. Virally supp. = virally suppressed = viral load less than 200 copies per ml on last viral load test. Diagnosed = people with diagnosed HIV living in Ontario = nominal HIV-positive diagnostic test and/or ≥1 viral load test, and not LTFU after 2 years. LTFU = lost to follow up = no record of a viral load test in more than 2 years, and no viral load test in later years. Individuals missing information on age were excluded (approximately 0.2%).

**Table O.** Number and percent of people newly diagnosed with HIV in Ontario who achieved virally suppressed within six months of diagnosis by age category, 2000 to 2014

| Year | ≤24 | | | 25-34 | | | 35-44 | | | 45-54 | | | ≥55 | | |
| --- | --- | --- | --- | --- | --- | --- | --- | --- | --- | --- | --- | --- | --- | --- | --- |
|  | Virally supp. | Newly diagnosed | % | Virally supp. | Newly diagnosed | % | Virally supp. | Newly diagnosed | % | Virally supp. | Newly diagnosed | % | Virally supp. | Newly diagnosed | % |
| 2000 -2002 | 22 | 146 | 15.1% | 90 | 468 | 19.2% | 117 | 458 | 25.5% | 40 | 155 | 25.8% | 24 | 86 | 27.9% |
| 2002-2005 | 21 | 162 | 13.0% | 100 | 541 | 18.5% | 115 | 620 | 18.5% | 61 | 246 | 24.8% | 25 | 108 | 23.1% |
| 2006-2008 | 33 | 203 | 16.3% | 86 | 524 | 16.4% | 138 | 565 | 24.4% | 76 | 319 | 23.8% | 28 | 111 | 25.2% |
| 2009-2011 | 33 | 204 | 16.2% | 102 | 455 | 22.4% | 139 | 449 | 31.0% | 100 | 317 | 31.5% | 52 | 149 | 34.9% |
| 2012-2014 | 67 | 175 | 38.3% | 167 | 436 | 38.3% | 127 | 336 | 37.8% | 118 | 260 | 45.4% | 60 | 128 | 46.9% |

**Notes:** Data provided by the Public Health Ontario Laboratory. See manuscript for more details on indicator definitions. Percentages calculated using ‘virall supp.’ in the numerator and ‘newly diagnosed’ in the denominator. Virally supp. = virally suppressed within 6 months of diagnosis = record of a viral load less than 200 copies/ml within 6 months of diagnosis. Newly diagnosed = nominal HIV-positive diagnostic test and no evidence of previous diagnosis (i.e. no detectable viral load test or CD4 count before their diagnosis date, and first VL after diagnosis not suppressed). Percents aggregated over multiple years to reduce year-to-year variation due to small counts. The year 2015 not included as some individuals diagnosed in that year would not have had time to reach viral suppression. Individuals missing information on age were excluded (approximately 0.2%).
