# Supplementary material for: Trends in HIV care cascade engagement among diagnosed people living with HIV in Ontario, Canada: A retrospective, population-based cohort study
Source: PLoS One. 2019 Jan 4;14(1):e0210096. doi: 10.1371/journal.pone.0210096 (PMC6319701; doi:10.1371/journal.pone.0210096)
Supplement: S2 Supporting Information — (DOCX) [file pone.0210096.s005.docx]

**Fig A. Flow diagram for the creation of the newly diagnosed sample from the Public Health Ontario Laboratory HIV datamart.** Evidence of previous diagnosis = 1) record of a detectable VL test or CD4 count before diagnosis or 2) first VL test after diagnosis was suppressed. VL = viral load. Unless otherwise noted, date of HIV diagnostic and VL testing based on day the test was *received* at the laboratory for testing (as opposed to date the result was *reported* by the laboratory to the ordering provider). Data provided by Public Health Ontario Laboratory.


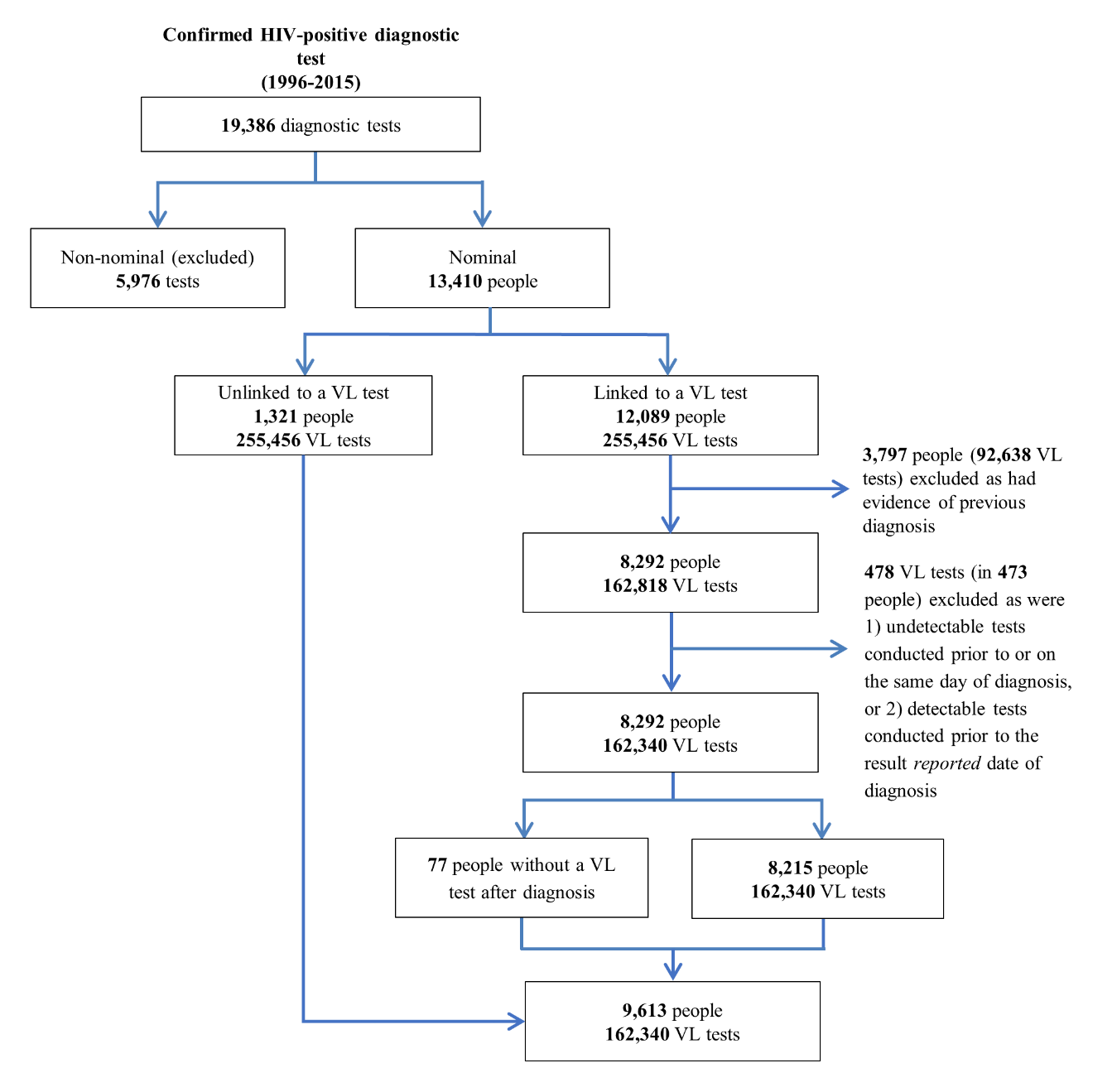


**Table A.** Number of individuals newly diagnosed with HIV in Ontario, Public Health Ontario Laboratory HIV datamart, 2000 to 2015.

| Year | Newly diagnosed |
| --- | --- |
| 2000 | 368 |
| 2001 | 424 |
| 2002 | 542 |
| 2003 | 553 |
| 2004 | 570 |
| 2005 | 567 |
| 2006 | 599 |
| 2007 | 555 |
| 2008 | 574 |
| 2009 | 523 |
| 2010 | 530 |
| 2011 | 528 |
| 2012 | 450 |
| 2013 | 415 |
| 2014 | 473 |
| 2015 | 502 |

Note: Data provided by Public Health Ontario Laboratory. Newly diagnosed = individuals with a nominal HIV-positive diagnostic test and no evidence of previous diagnosis. See manuscript for more details on inclusion/exclusion criteria. These numbers underestimate the actual number of individuals newly diagnosed in Ontario as individuals diagnosed non-nominally are excluded unless they also receive a nominal diagnostic test.
