# Supplementary material for: Trends in HIV care cascade engagement among diagnosed people living with HIV in Ontario, Canada: A retrospective, population-based cohort study
Source: PLoS One. 2019 Jan 4;14(1):e0210096. doi: 10.1371/journal.pone.0210096 (PMC6319701; doi:10.1371/journal.pone.0210096)
Supplement: S3 Supporting Information — (DOCX) [file pone.0210096.s006.docx]

**Table A.** Number and percent of people with diagnosed HIV living in Ontario who were in care by sex, 2000 to 2015

| **Year** | **Male** | | | **Female** | | |
| --- | --- | --- | --- | --- | --- | --- |
|  | In care  (numerator) | Diagnosed (denominator) | % | In care  (numerator) | Diagnosed (denominator) | % |
|  | At least 1 VL test | Nominal HIV-positive diagnostic test and/or ≥1 VL test, and not LTFU after 2 years |  | At least 1 VL test | Nominal HIV-positive diagnostic test and/or ≥1 VL test, and not LTFU after 2 years |  |
| **2000** | 6,179 | 7,511 | 82.3% | 1,024 | 1,328 | 77.1% |
| **2001** | 6,481 | 7,877 | 82.3% | 1,188 | 1,485 | 80.0% |
| **2002** | 6,928 | 8,295 | 83.5% | 1,304 | 1,646 | 79.2% |
| **2003** | 7,294 | 8,715 | 83.7% | 1,481 | 1,854 | 79.9% |
| **2004** | 7,710 | 9,115 | 84.6% | 1,625 | 2,003 | 81.1% |
| **2005** | 8,059 | 9,504 | 84.8% | 1,782 | 2,155 | 82.7% |
| **2006** | 8,418 | 9,940 | 84.7% | 1,981 | 2,367 | 83.7% |
| **2007** | 8,714 | 10,293 | 84.7% | 2,099 | 2,529 | 83.0% |
| **2008** | 9,123 | 10,687 | 85.4% | 2,193 | 2,680 | 81.8% |
| **2009** | 9,407 | 11,010 | 85.4% | 2,309 | 2,796 | 82.6% |
| **2010** | 9,656 | 11,302 | 85.4% | 2,327 | 2,831 | 82.2% |
| **2011** | 9,983 | 11,634 | 85.8% | 2,481 | 2,931 | 84.6% |
| **2012** | 10,209 | 11,833 | 86.3% | 2,555 | 3,011 | 84.9% |
| **2013** | 10,528 | 12,112 | 86.9% | 2,625 | 3,089 | 85.0% |
| **2014** | 10,862 | 12,406 | 87.6% | 2,680 | 3,146 | 85.2% |
| **2015** | 11,173 | 12,724 | 87.8% | 2,793 | 3,257 | 85.8% |

**Notes:** Data provided by the Public Health Ontario Laboratory. See manuscript for more information on indicator definitions. Diagnosed = people with diagnosed HIV living in Ontario. LTFU = lost to follow up = no record of a VL test in more than 2 years and no VL test in later years. VL = viral load. Individuals missing information on sex were excluded (approximately 0.4%).

**Table B.** Number and percent of people with diagnosed HIV living in Ontario who were on ART by sex, 2000 to 2015

| **Year** | **Male** | | | **Female** | | |
| --- | --- | --- | --- | --- | --- | --- |
|  | On ART  (numerator) | Diagnosed (denominator) | % | On ART  (numerator) | Diagnosed (denominator) | % |
|  | Documented on ART, or ART status missing and virally suppressed, on last VL test | Nominal HIV-positive diagnostic test and/or ≥1 VL test, and not LTFU after 2 years |  | Documented on ART, or ART status missing and virally suppressed, on last VL test | Nominal HIV-positive diagnostic test and/or ≥1 VL test, and not LTFU after 2 years |  |
| **2000** | 4,222 | 7,511 | 56.2% | 658 | 1,328 | 49.5% |
| **2001** | 4,421 | 7,877 | 56.1% | 757 | 1,485 | 51.0% |
| **2002** | 4,731 | 8,295 | 57.0% | 824 | 1,646 | 50.1% |
| **2003** | 5,017 | 8,715 | 57.6% | 915 | 1,854 | 49.4% |
| **2004** | 5,362 | 9,115 | 58.8% | 1,022 | 2,003 | 51.0% |
| **2005** | 5,729 | 9,504 | 60.3% | 1,127 | 2,155 | 52.3% |
| **2006** | 6,138 | 9,940 | 61.8% | 1,294 | 2,367 | 54.7% |
| **2007** | 6,562 | 10,293 | 63.8% | 1,440 | 2,529 | 56.9% |
| **2008** | 7,182 | 10,687 | 67.2% | 1,586 | 2,680 | 59.2% |
| **2009** | 7,635 | 11,010 | 69.3% | 1,759 | 2,796 | 62.9% |
| **2010** | 8,047 | 11,302 | 71.2% | 1,855 | 2,831 | 65.5% |
| **2011** | 8,568 | 11,634 | 73.6% | 2,032 | 2,931 | 69.3% |
| **2012** | 9,020 | 11,833 | 76.2% | 2,193 | 3,011 | 72.8% |
| **2013** | 9,553 | 12,112 | 78.9% | 2,283 | 3,089 | 73.9% |
| **2014** | 9,978 | 12,406 | 80.4% | 2,386 | 3,146 | 75.8% |
| **2015** | 10,437 | 12,724 | 82.0% | 2,532 | 3,257 | 77.7% |

**Notes:** Data provided by the Public Health Ontario Laboratory. See manuscript for more information on indicator definitions. Diagnosed = people with diagnosed HIV living in Ontario. LTFU = lost to follow up = no record of a VL test in more than 2 years, and no VL test in later years. VL = viral load. ART = antiretroviral treatment. ART status documented on VL test requisition by ordering provider and missing for 17-20% of requisitions. Individuals missing information on sex were excluded (approximately 0.4%).

**Table C.** Number and percent of people with diagnosed HIV living in Ontario on ART who were virally suppressed by sex, 2000 to 2015

| **Year** | **Male** | | | **Female** | | |
| --- | --- | --- | --- | --- | --- | --- |
|  | Virally suppressed  (numerator) | On ART  (denominator) | % | Virally suppressed  (numerator) | On ART  (denominator) | % |
|  | VL less than 200 copies per ml on last VL test, and known on ART or ART status missing, on last VL test | Known on ART, or ART status missing, on last VL test |  | VL less than 200 copies per ml on last VL test, and known on ART or ART status missing, on last VL test | Known on ART, or ART status missing, on last VL test |  |
| **2000** | 3,039 | 4,798 | 63.3% | 456 | 732 | 62.3% |
| **2001** | 3,375 | 5,043 | 66.9% | 546 | 845 | 64.6% |
| **2002** | 3,711 | 5,405 | 68.7% | 629 | 905 | 69.5% |
| **2003** | 4,073 | 5,670 | 71.8% | 737 | 1,031 | 71.5% |
| **2004** | 4,577 | 6,061 | 75.5% | 836 | 1,158 | 72.2% |
| **2005** | 4,970 | 6,391 | 77.8% | 954 | 1,267 | 75.3% |
| **2006** | 5,497 | 6,840 | 80.4% | 1,123 | 1,408 | 79.8% |
| **2007** | 6,041 | 7,178 | 84.2% | 1,294 | 1,580 | 81.9% |
| **2008** | 6,725 | 7,752 | 86.8% | 1,444 | 1,747 | 82.7% |
| **2009** | 7,226 | 8,148 | 88.7% | 1,618 | 1,884 | 85.9% |
| **2010** | 7,642 | 8,482 | 90.1% | 1,706 | 1,968 | 86.7% |
| **2011** | 8,213 | 8,974 | 91.5% | 1,857 | 2,130 | 87.2% |
| **2012** | 8,644 | 9,388 | 92.1% | 2,049 | 2,299 | 89.1% |
| **2013** | 9,202 | 9,884 | 93.1% | 2,132 | 2,395 | 89.0% |
| **2014** | 9,648 | 10,264 | 94.0% | 2,264 | 2,470 | 91.7% |
| **2015** | 10,128 | 10,697 | 94.7% | 2,430 | 2,601 | 93.4% |

**Notes:** Data provided by the Public Health Ontario Laboratory. See manuscript for more information on indicator definitions. LTFU = no record of a VL test in more than 2 years, and no VL test in later years. VL = viral load. ART = antiretroviral treatment. ART status documented on VL test requisition by ordering provider and missing for 17-20% of requisitions. Individuals missing information on sex were excluded (approximately 0.4%).

**Table D.** Number and percent of people newly diagnosed with HIV in Ontario who linked to care within three months by sex, 2000 to 2014

| **Year** | **Male** | | | **Female** | | |
| --- | --- | --- | --- | --- | --- | --- |
|  | Linked to care  (numerator) | Newly diagnosed  (denominator) | % | Linked to care  (numerator) | Newly diagnosed  (denominator) | % |
|  | First VL within 3 months of HIV diagnosis | Nominal HIV-positive diagnostic test and no evidence of previous diagnosis |  | First VL within 3 months of HIV diagnosis | Nominal HIV-positive diagnostic test and no evidence of previous diagnosis |  |
| **2000** | 176 | 264 | 66.7% | 72 | 102 | 70.6% |
| **2001** | 191 | 284 | 67.3% | 83 | 138 | 60.1% |
| **2002** | 262 | 361 | 72.6% | 121 | 178 | 68.0% |
| **2003** | 272 | 367 | 74.1% | 125 | 182 | 68.7% |
| **2004** | 311 | 396 | 78.5% | 130 | 172 | 75.6% |
| **2005** | 312 | 419 | 74.5% | 104 | 146 | 71.2% |
| **2006** | 324 | 408 | 79.4% | 139 | 188 | 73.9% |
| **2007** | 307 | 402 | 76.4% | 108 | 150 | 72.0% |
| **2008** | 304 | 416 | 73.1% | 102 | 153 | 66.7% |
| **2009** | 301 | 399 | 75.4% | 100 | 122 | 82.0% |
| **2010** | 329 | 409 | 80.4% | 88 | 114 | 77.2% |
| **2011** | 325 | 401 | 81.0% | 99 | 122 | 81.1% |
| **2012** | 280 | 337 | 83.1% | 88 | 109 | 80.7% |
| **2013** | 284 | 335 | 84.8% | 64 | 76 | 84.2% |
| **2014** | 309 | 371 | 83.3% | 75 | 96 | 78.1% |

**Notes:** Data provided by the Public Health Ontario Laboratory. See manuscript for more information on indicator definitions. VL = viral load. No evidence of previous diagnosis = no detectable viral load test or CD4 count before diagnosis date, and first VL after diagnosis not virally suppressed. The year 2015 not included as some individuals diagnosed in this year would not have had time to link to care. Individuals missing information on sex were excluded (approximately 0.4%).

**Table E.** Number and percent of people with diagnosed HIV living in Ontario who were in care by age category, 2000 to 2015

| Year | ≤24 | | | 25-34 | | | 35-44 | | | 45-54 | | | ≥55 | | |
| --- | --- | --- | --- | --- | --- | --- | --- | --- | --- | --- | --- | --- | --- | --- | --- |
|  | In care | Diagnosed | % | In care | Diagnosed | % | In care | Diagnosed | % | In care | Diagnosed | % | In care | Diagnosed | % |
| 2000 | 252 | 317 | 79.5% | 1,462 | 1,910 | 76.5% | 3,334 | 4,005 | 83.2% | 1,611 | 1,899 | 84.8% | 535 | 662 | 80.8% |
| 2001 | 284 | 343 | 82.8% | 1,407 | 1,811 | 77.7% | 3,558 | 4,297 | 82.8% | 1,766 | 2,083 | 84.8% | 647 | 785 | 82.4% |
| 2002 | 287 | 356 | 80.6% | 1,399 | 1,784 | 78.4% | 3,785 | 4,546 | 83.3% | 2,001 | 2,307 | 86.7% | 758 | 915 | 82.8% |
| 2003 | 302 | 370 | 81.6% | 1,395 | 1,771 | 78.8% | 3,956 | 4,780 | 82.8% | 2,231 | 2,574 | 86.7% | 890 | 1,050 | 84.8% |
| 2004 | 327 | 386 | 84.7% | 1,430 | 1,776 | 80.5% | 4,068 | 4,833 | 84.2% | 2,494 | 2,898 | 86.1% | 1,017 | 1,208 | 84.2% |
| 2005 | 345 | 411 | 83.9% | 1,423 | 1,753 | 81.2% | 4,154 | 4,914 | 84.5% | 2,769 | 3,212 | 86.2% | 1,155 | 1,366 | 84.6% |
| 2006 | 359 | 429 | 83.7% | 1,476 | 1,832 | 80.6% | 4,122 | 4,924 | 83.7% | 3,095 | 3,582 | 86.4% | 1,347 | 1,538 | 87.6% |
| 2007 | 362 | 429 | 84.4% | 1,456 | 1,826 | 79.7% | 4,019 | 4,811 | 83.5% | 3,447 | 4,006 | 86.0% | 1,530 | 1,750 | 87.4% |
| 2008 | 370 | 445 | 83.1% | 1,464 | 1,867 | 78.4% | 3,885 | 4,639 | 83.7% | 3,859 | 4,432 | 87.1% | 1,737 | 1,983 | 87.6% |
| 2009 | 381 | 450 | 84.7% | 1,492 | 1,872 | 79.7% | 3,678 | 4,408 | 83.4% | 4,226 | 4,865 | 86.9% | 1,941 | 2,215 | 87.6% |
| 2010 | 388 | 445 | 87.2% | 1,560 | 1,889 | 82.6% | 3,480 | 4,178 | 83.3% | 4,493 | 5,221 | 86.1% | 2,169 | 2,507 | 86.5% |
| 2011 | 399 | 467 | 85.4% | 1,592 | 1,905 | 83.6% | 3,338 | 4,001 | 83.4% | 4,765 | 5,477 | 87.0% | 2,476 | 2,822 | 87.7% |
| 2012 | 390 | 460 | 84.8% | 1,585 | 1,912 | 82.9% | 3,263 | 3,866 | 84.4% | 4,875 | 5,600 | 87.1% | 2,752 | 3,121 | 88.2% |
| 2013 | 381 | 441 | 86.4% | 1,597 | 1,942 | 82.2% | 3,164 | 3,716 | 85.1% | 5,013 | 5,727 | 87.5% | 3,083 | 3,480 | 88.6% |
| 2014 | 375 | 438 | 85.6% | 1,658 | 1,983 | 83.6% | 3,059 | 3,596 | 85.1% | 5,084 | 5,779 | 88.0% | 3,452 | 3,861 | 89.4% |
| 2015 | 400 | 469 | 85.3% | 1,644 | 2,009 | 81.8% | 3,012 | 3,529 | 85.3% | 5,092 | 5,737 | 88.8% | 3,898 | 4,329 | 90.0% |

**Notes:** Data provided by the Public Health Ontario Laboratory. See manuscript for more information on indicator definitions. Percentages calculated using ‘in care’ in the numerator and ‘diagnosed’ in the denominator. In care = At least one viral test in a given year. Diagnosed = people with diagnosed HIV living in Ontario = nominal HIV-positive diagnostic test and/or ≥1 VL test, and not LTFU after 2 years. LTFU = lost to follow up = no record of a VL test in more than 2 years, and no viral load test in later years. Individuals missing information on age were excluded (approximately 0.2%).

**Table F.** Number and percent of people with diagnosed HIV living in Ontario who were on ART by age category, 2000 to 2015

| Year | ≤24 | | | 25-34 | | | 35-44 | | | 45-54 | | | ≥55 | | |
| --- | --- | --- | --- | --- | --- | --- | --- | --- | --- | --- | --- | --- | --- | --- | --- |
|  | On ART | Diagnosed | % | On ART | Diagnosed | % | On ART | Diagnosed | % | On ART | Diagnosed | % | On ART | Diagnosed | % |
| 2000 | 157 | 317 | 49.5% | 833 | 1,910 | 43.6% | 2,276 | 4,005 | 56.8% | 1,194 | 1,899 | 62.9% | 415 | 662 | 62.7% |
| 2001 | 159 | 343 | 46.4% | 748 | 1,811 | 41.3% | 2,429 | 4,297 | 56.5% | 1,334 | 2,083 | 64.0% | 507 | 785 | 64.6% |
| 2002 | 164 | 356 | 46.1% | 746 | 1,784 | 41.8% | 2,559 | 4,546 | 56.3% | 1,486 | 2,307 | 64.4% | 599 | 915 | 65.5% |
| 2003 | 176 | 370 | 47.6% | 683 | 1,771 | 38.6% | 2,653 | 4,780 | 55.5% | 1,684 | 2,574 | 65.4% | 736 | 1,050 | 70.1% |
| 2004 | 176 | 386 | 45.6% | 698 | 1,776 | 39.3% | 2,747 | 4,833 | 56.8% | 1,923 | 2,898 | 66.4% | 841 | 1,208 | 69.6% |
| 2005 | 162 | 411 | 39.4% | 683 | 1,753 | 39.0% | 2,872 | 4,914 | 58.4% | 2,167 | 3,212 | 67.5% | 976 | 1,366 | 71.4% |
| 2006 | 173 | 429 | 40.3% | 721 | 1,832 | 39.4% | 2,927 | 4,924 | 59.4% | 2,445 | 3,582 | 68.3% | 1,163 | 1,538 | 75.6% |
| 2007 | 185 | 429 | 43.1% | 759 | 1,826 | 41.6% | 2,899 | 4,811 | 60.3% | 2,816 | 4,006 | 70.3% | 1,344 | 1,750 | 76.8% |
| 2008 | 192 | 445 | 43.1% | 801 | 1,867 | 42.9% | 2,966 | 4,639 | 63.9% | 3,240 | 4,432 | 73.1% | 1,569 | 1,983 | 79.1% |
| 2009 | 222 | 450 | 49.3% | 889 | 1,872 | 47.5% | 2,861 | 4,408 | 64.9% | 3,630 | 4,865 | 74.6% | 1,792 | 2,215 | 80.9% |
| 2010 | 245 | 445 | 55.1% | 993 | 1,889 | 52.6% | 2,796 | 4,178 | 66.9% | 3,954 | 5,221 | 75.7% | 2,002 | 2,507 | 79.9% |
| 2011 | 242 | 467 | 51.8% | 1,082 | 1,905 | 56.8% | 2,775 | 4,001 | 69.4% | 4,255 | 5,477 | 77.7% | 2,323 | 2,822 | 82.3% |
| 2012 | 259 | 460 | 56.3% | 1,205 | 1,912 | 63.0% | 2,778 | 3,866 | 71.9% | 4,447 | 5,600 | 79.4% | 2,604 | 3,121 | 83.4% |
| 2013 | 268 | 441 | 60.8% | 1,283 | 1,942 | 66.1% | 2,793 | 3,716 | 75.2% | 4,637 | 5,727 | 81.0% | 2,932 | 3,480 | 84.3% |
| 2014 | 276 | 438 | 63.0% | 1,373 | 1,983 | 69.2% | 2,718 | 3,596 | 75.6% | 4,774 | 5,779 | 82.6% | 3,300 | 3,861 | 85.5% |
| 2015 | 320 | 469 | 68.2% | 1,409 | 2,009 | 70.1% | 2,756 | 3,529 | 78.1% | 4,825 | 5,737 | 84.1% | 3,737 | 4,329 | 86.3% |

**Notes:** Data provided by the Public Health Ontario Laboratory. See manuscript for more information on indicator definitions. Percentages calculated using ‘on ART’ in the numerator and ‘diagnosed’ in the denominator. On ART = documented on ART, or ART status missing and virally suppressed, on last viral load test. On ART documented by ordering provider on viral load test requisitions and missing for 17-20% of requisitions. Diagnosed = people with diagnosed HIV living in Ontario = nominal HIV-positive diagnostic test and/or ≥1 viral load test, and not LTFU after 2 years. LTFU = lost to follow up = no record of a viral load test in more than 2 years, and no viral load test in later years. Individuals missing information on age were excluded (approximately 0.2%).

**Table G.** Number and percent of people with diagnosed HIV living in Ontario on ART who were virally suppressed by age category, 2000 to 2015

| Year | ≤24 | | | 25-34 | | | 35-44 | | | 45-54 | | | ≥55 | | |
| --- | --- | --- | --- | --- | --- | --- | --- | --- | --- | --- | --- | --- | --- | --- | --- |
|  | Virally  Supp. | On ART | % | Virally  Supp. | On ART | % | Virally  Supp. | On ART | % | Virally  Supp. | On ART | % | Virally  Supp. | On ART | % |
| 2000 | 77 | 173 | 44.5% | 609 | 998 | 61.0% | 1,649 | 2,584 | 63.8% | 840 | 1,317 | 63.8% | 316 | 450 | 70.2% |
| 2001 | 93 | 179 | 52.0% | 563 | 919 | 61.3% | 1,840 | 2,773 | 66.4% | 1,015 | 1,460 | 69.5% | 409 | 552 | 74.1% |
| 2002 | 107 | 190 | 56.3% | 570 | 905 | 63.0% | 1,994 | 2,923 | 68.2% | 1,179 | 1,641 | 71.8% | 489 | 649 | 75.3% |
| 2003 | 114 | 198 | 57.6% | 546 | 869 | 62.8% | 2,153 | 3,021 | 71.3% | 1,372 | 1,824 | 75.2% | 625 | 789 | 79.2% |
| 2004 | 118 | 210 | 56.2% | 576 | 903 | 63.8% | 2,325 | 3,110 | 74.8% | 1,647 | 2,100 | 78.4% | 748 | 897 | 83.4% |
| 2005 | 111 | 193 | 57.5% | 578 | 868 | 66.6% | 2,451 | 3,218 | 76.2% | 1,894 | 2,341 | 80.9% | 893 | 1,041 | 85.8% |
| 2006 | 137 | 206 | 66.5% | 622 | 908 | 68.5% | 2,571 | 3,280 | 78.4% | 2,205 | 2,629 | 83.9% | 1,082 | 1,222 | 88.5% |
| 2007 | 151 | 219 | 68.9% | 658 | 937 | 70.2% | 2,641 | 3,213 | 82.2% | 2,611 | 2,986 | 87.4% | 1,275 | 1,404 | 90.8% |
| 2008 | 161 | 234 | 68.8% | 720 | 969 | 74.3% | 2,719 | 3,249 | 83.7% | 3,050 | 3,423 | 89.1% | 1,519 | 1,624 | 93.5% |
| 2009 | 190 | 260 | 73.1% | 795 | 1,024 | 77.6% | 2,660 | 3,095 | 85.9% | 3,455 | 3,806 | 90.8% | 1,744 | 1,848 | 94.4% |
| 2010 | 213 | 273 | 78.0% | 913 | 1,124 | 81.2% | 2,591 | 2,980 | 86.9% | 3,760 | 4,117 | 91.3% | 1,953 | 2,054 | 95.1% |
| 2011 | 212 | 281 | 75.4% | 994 | 1,192 | 83.4% | 2,601 | 2,938 | 88.5% | 4,064 | 4,415 | 92.0% | 2,270 | 2,369 | 95.8% |
| 2012 | 226 | 294 | 76.9% | 1,121 | 1,318 | 85.1% | 2,606 | 2,929 | 89.0% | 4,256 | 4,574 | 93.0% | 2,562 | 2,662 | 96.2% |
| 2013 | 235 | 303 | 77.6% | 1,199 | 1,380 | 86.9% | 2,644 | 2,914 | 90.7% | 4,458 | 4,787 | 93.1% | 2,873 | 2,974 | 96.6% |
| 2014 | 251 | 304 | 82.6% | 1,308 | 1,449 | 90.3% | 2,583 | 2,837 | 91.0% | 4,607 | 4,883 | 94.3% | 3,237 | 3,341 | 96.9% |
| 2015 | 293 | 349 | 84.0% | 1,336 | 1,486 | 89.9% | 2,654 | 2,845 | 93.3% | 4,672 | 4,908 | 95.2% | 3,680 | 3,789 | 97.1% |

**Notes:** Data provided by the Public Health Ontario Laboratory. See manuscript for more information on indicator definitions. Percentages calculated using ‘virally supp.’ in the numerator and ‘on ART’ in the denominator. Virally supp. = virally suppressed = viral load less than 200 copies per ml on last viral load test. On ART = documented on ART, or ART status missing, on last viral load test. On ART documented by ordering provider on viral load test requisitions and is missing for 17-20% of requisitions. Individuals missing information on age were excluded (approximately 0.2%).

**Table H.** Number and percent of people newly diagnosed with HIV in Ontario who linked to care within three months of diagnosis by age category, 2000 to 2014

| Year | ≤24 | | | 25-34 | | | 35-44 | | | 45-54 | | | ≥55 | | |
| --- | --- | --- | --- | --- | --- | --- | --- | --- | --- | --- | --- | --- | --- | --- | --- |
|  | Linked to care | Newly diagnosed | % | Linked to care | Newly diagnosed | % | Linked to care | Newly diagnosed | % | Linked to care | Newly diagnosed | % | Linked to care | Newly diagnosed | % |
| 2000 -2002 | 100 | 146 | 68.5% | 320 | 468 | 68.4% | 321 | 458 | 70.1% | 106 | 155 | 68.4% | 60 | 86 | 69.8% |
| 2002-2005 | 114 | 162 | 70.4% | 394 | 541 | 72.8% | 477 | 620 | 76.9% | 192 | 246 | 78.0% | 80 | 108 | 74.1% |
| 2006-2008 | 154 | 203 | 75.9% | 372 | 524 | 71.0% | 423 | 565 | 74.9% | 253 | 319 | 79.3% | 86 | 111 | 77.5% |
| 2009-2011 | 150 | 204 | 73.5% | 352 | 455 | 77.4% | 371 | 449 | 82.6% | 253 | 317 | 79.8% | 123 | 149 | 82.6% |
| 2012-2014 | 145 | 175 | 82.9% | 347 | 436 | 79.6% | 284 | 336 | 84.5% | 223 | 260 | 85.8% | 108 | 128 | 84.4% |

**Notes:** Data provided by the Public Health Ontario Laboratory. See manuscript for more information on indicator definitions. Percentages calculated using ‘linked to care’ in the numerator and ‘newly diagnosed’ in the denominator. Linked to care = first VL within three months of HIV diagnosis. Newly diagnosed = nominal HIV-positive diagnostic test and no evidence of previous diagnosis (i.e. no detectable viral load test or CD4 count before diagnosis date, and first VL after diagnosis not suppressed). Percents aggregated over multiple years to reduce year-to-year variation due to small counts. The year 2015 not included as some individuals diagnosed in this year would not have had time to link to care. Individuals missing information on age were excluded (approximately 0.2%).
